# SCO2, LYN, and FGR as Mitochondria-Associated Immune-Inflammatory Candidate Signatures Across PMOS and MCI Datasets

**DOI:** 10.3390/biomedicines14102205

**Published:** 2026-09-29

**Authors:** Lu Wang, Zhijing Tang, Hao Zhu, Hailin Yu, Xiaohong Xue, Wei Zhang

**Affiliations:** 1 Department of Reproductive Endocrinology, Obstetrics and Gynecology Hospital, Fudan University, 413 Zhaozhou Road, Shanghai 200011, China; 15111250007@fudan.edu.cn (L.W.);; 2 Obstetrics and Gynecology Hospital, Fudan University, 413 Zhaozhou Road, Shanghai 200011, China; 3 Shanghai Key Laboratory of Female Reproductive Endocrine Related Disease, 413 Zhaozhou Road, Shanghai 200011, China

**Keywords:** mild cognitive impairment, mitochondria, machine learning, polyendocrine metabolic ovarian syndrome, single-cell RNA sequencing

## Abstract

**Background**: Polyendocrine metabolic ovarian syndrome (PMOS), previously known as polycystic ovary syndrome (PCOS), is a prevalent endocrine disorder increasingly associated with mild cognitive impairment (MCI). Mitochondrial dysfunction and immune dysregulation may underlie this association; however, shared molecular features between these conditions remain systematically uninvestigated. **Methods**: We used PMOS (granulosa cell, *n* = 16) and MCI (peripheral blood, *n* = 243) to identify differentially expressed genes (DEGs) intersecting mitochondrial-related genes (MRGs), supported by enrichment and prioritized by machine-learning with cross-validation. Boxplots in PMOS and MCI confirmed directionality. Single-cell RNA sequencing (scRNA-seq) identified cell types and communication. In vitro experiments assessed SCO2, LYN, and FGR in cell lines. **Results**: We identified 16 mitochondrial-related genes shared by PMOS and MCI datasets. Enrichment implicated NF-κB and TNF signaling in PMOS/MCI immunometabolic features. SCO2, LYN, and FGR were upregulated across datasets, although LYN was discordant in granulosa comparison. Single-cell sequencing identified five subtypes. Pseudotime showed SCO2 and LYN upregulation in early/late stages, whereas FGR was undetectable. Secondary annotation identified plasmacytoid dendritic cells, classical monocytes, and pro-B cells; pro-B cells predominated in controls (47.5%) and classical monocytes in disease (40.5%). In vitro validation showed mRNA/protein upregulation of SCO2, LYN, and FGR in PMOS/MCI models versus control. **Conclusions**: This study identified SCO2, LYN, and FGR as mitochondria-associated immune-inflammatory signatures in PMOS and MCI datasets. Differential expression, feature-ranking models, pathway enrichment, transcriptional deconvolution, single-cell contextualization, and cell assessment support were utilized. Without PMOS–MCI cohorts and functional perturbation experiments, findings remain hypothesis-generating, unvalidated biomarkers or causal mechanisms.

## 1. Introduction

Polyendocrine metabolic ovarian syndrome (PMOS), formerly polycystic ovary syndrome (PCOS), is an endocrine–metabolic disorder affecting 6–20% of women of reproductive age [1,2,3]. PMOS features inflammation, immune changes, and metabolic complications [4,5]. Insulin resistance (IR) has been a key pathophysiological feature in PMOS since the 1980s [6]. Studies show that polycystic ovarian morphology (PCOM), including phenotypes and increased follicle number, is associated with insulin-resistance indices including homeostatic model assessment for insulin resistance (HOMA-IR) and quantitative insulin-sensitivity check index (QUICKI), and IR contributes to granulosa-cell functional perturbations in patients [7,8]. PMOS is comorbid with mild cognitive impairment (MCI), with memory, executive, and visuospatial deficits [9]. Evidence indicates that women with PMOS exhibit cognitive alterations mainly in memory performance; these are distinct from age-related cognitive decline yet are incompletely characterized at the molecular level [10]. Disturbances such as insulin resistance and pro-inflammatory cytokines may impair neural function by disrupting the blood–brain barrier, promoting neuroinflammation, and altering hippocampal and prefrontal plasticity [11]. This suggests that PMOS may predispose women to neurocognitive decline [12,13]. However, studies on biomarkers and mechanisms linking PMOS to cognitive decline are limited.

Mitochondrial dysfunction may mechanistically link PMOS and MCI. In PMOS, mitochondrial abnormalities, such as impaired oxidative phosphorylation (OXPHOS), excessive reactive oxygen species (ROS) production, and mitochondrial DNA (mtDNA) instability, contribute to systemic metabolic dysregulation [14,15]. Similarly, MCI involves cerebral energy deficits, oxidative stress, and neuroinflammatory cascades, indicating mitochondrial dysfunction in the brain [16]. PMOS-related systemic factors may worsen mitochondrial dysfunction and increase metabolic stress in the central nervous system, thereby accelerating cognitive decline [17]. Despite this evidence, no study has systematically investigated mitochondrial-related genes (MRGs) or developed diagnostic models for PMOS–MCI.

However, evidence linking PMOS to MCI remains inconclusive. Several studies detected cognitive differences between PMOS patients and controls after adjustment for body mass index (BMI) and metabolic confounders [18,19,20], but direction and magnitude vary by design and domain. Thus, cross-dataset correlations do not establish causality or linkage. We compared PMOS granulosa-cell and MCI blood datasets. We did not compare transcriptomic profiles of granulosa and peripheral blood mononuclear cells or use granulosa cells as a blood-cell surrogate. Instead, we extracted differentially expressed genes from each disease’s relevant tissue and screened overlapping MRGs. Granulosa cells are a key PMOS target tissue with well-documented mitochondrial dysfunction [21]; because brain tissue is difficult to obtain in MCI research, peripheral blood mononuclear cells serve as systemic biosensors of immunometabolic and mitochondrial status [14]. Concordant changes may indicate conserved pathogenic signals. This cross-tissue strategy, used in PMOS–NAFLD [22] and PMOS–T2DM [23] studies, identifies pathway-level signatures, not tissue-specific biomarkers.

Studies have characterized mitochondrial alterations in conditions; however, the mitochondrial–immune transcriptional overlap between PMOS and MCI remains unexplored. Therefore, this cross-dataset transcriptomic analysis was designed instead of a comorbidity cohort investigation. By integrating PMOS granulosa cell and MCI peripheral blood transcriptomic datasets, mitochondria-related gene sets, machine-learning-based feature ranking, pathway-level analyses, and PMOS single-cell RNA-seq data, we aimed to identify candidate mitochondria-associated immune-inflammatory signatures that reflect convergent PMOS and MCI features. This provides a framework for mechanistic and clinical validation, rather than definitive causality or diagnostic utility.

## 2. Materials and Methods

### 2.1. Data Sources and Study Design

Polyendocrine metabolic ovarian syndrome (PMOS) and mild cognitive impairment (MCI) transcriptomic datasets retrieved from the Gene Expression Omnibus (GEO; http://www.ncbi.nlm.nih.gov/geo/, accessed on 5 August 2025) are summarized in Table S1. The PMOS discovery cohort integrated GSE34526 and GSE102293 granulosa samples, whereas GSE137684 served as the validation dataset. For MCI, GSE63061 was the discovery dataset and GSE63060 was the validation dataset; both were derived from peripheral blood. Given their origin from tissues and individuals rather than from a comorbid cohort, shared mitochondria-associated transcriptional signatures linking PMOS and MCI, rather than disease-specific markers, were identified. A PMOS single-cell RNA-sequencing (scRNA-seq) ovarian dataset from five patients and five controls was downloaded from Zenodo (https://zenodo.org/records/7942968, accessed on 5 August 2025) for context [24,25]. We obtained 1735 mitochondria-related genes (MRGs) from MitoCarta 3.0 (https://www.broadinstitute.org/mitocarta, accessed on 5 August 2025) [26].

### 2.2. Differential Expression Analysis and Identification of Shared Mitochondria-Associated Signatures

After batch effects between GSE34526 and GSE102293 were adjusted using the ComBat function from the R package sva (3.44.0) [27,28], the merged dataset served as the PMOS discovery cohort. ComBat correction was performed only when merging the two PMOS granulosa-cell datasets; no correction was applied to MCI discovery (GSE63061) or validation datasets (GSE137684, GSE63060). Differential expression analyses used limma (3.52.4) [29]. Given the small PMOS cohort (*n* = 16), PMOS-associated DEGs were screened using |log2FC| > 0.5 and nominal *p* < 0.05 to capture transcriptional changes. For the MCI discovery dataset (GSE63061, *n* = 243), we used adj.P.val < 0.05 without a log2FC cutoff to retain weak consistent signals. Both datasets used limma’s linear-model framework and empirical Bayes moderation. The PMOS *p*-values reflect limited power, precluding FDR correction and increasing false positives; intersection with MRGs and ML-based prioritization mitigated this. Sensitivity analyses should rank genes by Benjamini–Hochberg-adjusted *p*-value without an FDR claim, repeat screening with |log2FC| > 0.5, and analyze GSE34526 and GSE102293 separately. Genes were robust if direction was concordant in the PMOS discovery cohort and not contradictory in dataset-level sensitivity analysis. If unavailable, results are moved to limitations.

PMOS DEGs, MCI DEGs, and 1735 MitoCarta 3.0 mitochondria-related genes were intersected using ggVennDiagram (version 1.2.2) [30]. Upregulated and downregulated genes were intersected separately to retain concordant signals across the datasets. These genes were designated as shared mitochondria-associated signatures rather than PMOS–MCI comorbidity genes because the datasets came from different tissues and individuals.

### 2.3. Functional Enrichment and Pathway-Level Analyses

Gene Ontology (GO) and Kyoto Encyclopedia of Genes and Genomes (KEGG) enrichment analyses used the “clusterProfiler” R package (version 4.2.2) [31], with *p* < 0.05 significance. Protein–protein interactions among genes were explored using STRING with a confidence threshold of 0.4 as exploratory evidence. Spearman’s correlations between biomarkers and detectable genes in the PMOS and MCI datasets were calculated using the psych package to examine pathway-level dysregulation. Gene set enrichment analysis (GSEA) used c2.cp.kegg.v2023.2.Hs.symbols.gmt from MSigDB, with | normalized enrichment score| > 1 and adjusted *p* < 0.05. Gene set variation analysis (GSVA) and limma screened the enriched pathways using |t| > 2 and *p* < 0.05. Co-enriched pathways between PMOS and MCI were identified using Venn diagrams and visualized using Cytoscape (3.10.3).

Competing endogenous RNA (ceRNA), transcription factor (TF), disease gene, drug target, and docking analyses generated mechanistic hypotheses but not validation evidence. Biomarker–miRNA and miRNA–lncRNA interactions were obtained from starBase, transcription factor–biomarkers from ChEA3, and disease–gene relationships from the DisGeNET database. Drug–target interactions and docking analyses were used for in silico prioritization. Parameters, protein structures, ligand preparation, grid coordinates, scoring functions, and positive-control ligands are described in Supplementary Methods. To avoid overstating the data, ceRNA/TF/DisGeNET/docking outputs should be condensed into Supplementary Results or one supplementary figure, while the focus remains on transcriptomic, pathway, single-cell, and expression evidence. Parameters such as protein structures, grid coordinates, scoring functions, positive control ligands, and threshold status are documented in Table S2. These were labeled as hypothesis-generating.

### 2.4. Biomarker Prioritization and Exploratory Diagnostic Modeling

Candidate biomarkers were prioritized from the mitochondrial-associated gene set using the generalized linear model (GLM), random forest (RF), extreme gradient boosting (XGB), and support vector machine (SVM) algorithms. Residuals and importance were evaluated using “DALEX” (version 2.4.3) [32], and AUC via “auditor” (version 1.3.2). The algorithm with the lowest residuals and highest AUC was selected. Genes with importance scores > 20 were retained. A fixed random seed ensured reproducibility, and stratified k-fold cross-validation addressed the class imbalance. Due to the small PMOS sample size (*n* = 16), the outputs were interpreted as feature-ranking heuristics rather than validated models. Genes showing consistent directionality and significant differences across the PMOS and MCI datasets were retained. Exploratory nomograms were constructed using “rms” (version 6.8-0) [33]. Calibration curves were used to assess the agreement between predicted and observed probabilities via bootstrap resampling. Given the small sample size and lack of a confirmed PMOS–MCI cohort, these models are exploratory tools rather than clinically validated diagnostics. No external validation data were available.

### 2.5. Immune Infiltration and Regulatory Network Analyses

Immune-related patterns were estimated using Cell-type Identification by Estimating Relative Subsets of RNA Transcripts (CIBERSORT) (1.03) and the LM22 leukocyte gene signature matrix (LM22) matrix [34]. Because LM22 was developed for blood immune cell mixtures, its application to PMOS granulosa cell transcriptomes yielded CIBERSORT-derived outputs interpreted as immune-related transcriptional signatures rather than measurements of immune infiltration or cell abundance. For the MCI dataset, the CIBERSORT results were interpreted as immune cell composition estimates, while acknowledging the limitations of transcriptomic deconvolution.

Differences in immune-related signatures between disease and control groups were assessed by Wilcoxon rank-sum test, and associations of SCO2, LYN, and FGR with immune-related signatures were assessed by Spearman’s correlation. Testing corrections should be applied within the immune signature family, with exact and adjusted *p*-values reported when possible. For sensitivity analyses, gene set scores should be calculated using single-sample gene set enrichment analysis (ssGSEA) and/or xCell, and concordance with CIBERSORT-derived patterns is summarized in the Supplementary Results. If not completed, these should be stated as limitations and not as supporting evidence.

Relationships were explored by predicting biomarker–miRNA and miRNA–lncRNA interactions using starBase, transcription factor–biomarker interactions using ChEA3, and disease–gene associations using DisGeNET. Networks were visualized in Cytoscape using databases, evidence filters, node degree thresholds, and layout parameters (Supplementary Methods). The predicted networks were hypothesis-generating and not causal in nature.

### 2.6. Single-Cell RNA-Seq Analysis and Cellular-Context Validation

The PMOS single-cell RNA-seq dataset was processed using Seurat (5.1.0) [31]. Cells with <200 or >7500 detected genes or extreme counts were excluded, and genes in <3 cells were removed. Highly variable genes were identified using vst, principal components via JackStraw, clusters via FindNeighbors and FindClusters, and doublets using DoubletFinder. Cell types were annotated using canonical markers. To link single-cell states to bulk PMOS patterns, Scissor classifies cells as Scissor+, Scissor−, or Scissor0, based on bulk phenotype association. Differentially expressed genes between Scissor+ and Scissor− cells were identified using FindMarkers (log_2_FC ≥ 1.25, min.pct > 0.2, *p* < 0.05). Cell–cell communication was analyzed using CellPhoneDB. Candidate biomarker expression was visualized in violin plots; high-expression populations underwent pseudotime analysis using Monocle2 (2.26.0) [35]. Cell proportion statistics were summarized at the donor level to account for biological replication.

### 2.7. Cell Culture, Expression Validation, and Statistical Analysis

KGNs (Xiamen Yimo, Xiamen, China) were cultured in DMEM with 10% fetal bovine serum (FBS, Servicebio, G8003) and 1% penicillin/streptomycin(Servicebio, G4003). COV434 and BV2 cells (Zhengzhou Weisai) were maintained at 37 °C and 5% CO_2_. All cell lines were STR-authenticated and mycoplasma-free. BV2 cells were treated with 30 µg/mL lipopolysaccharide (LPS, MCE, HY-D1056) for 24 h to induce inflammation. The COV434/KGN comparison assessed whether elevated SCO2, LYN, and FGR expression in PMOS datasets occurred in granulosa cells, whereas the BV2 model evaluated these genes under neuroinflammatory conditions. RNA was isolated, assessed via NanoDrop-500 (Hangzhou Allsheng Instruments Co., Ltd., OD260/OD280 > 1.8), and reverse transcribed from 500 ng. Quantitative real-time PCR (qRT-PCR) used SYBR Green(ABclonal, Cat. No. RK21205) and GAPDH controls; SCO2, LYN, and FGR expression was calculated via the 2^−ΔΔCt^ method. Primer sequences, efficiency, and raw Ct values are in Table S3. Proteins were extracted using RIPA buffer(Beyotime, Cat. No. P0013B), quantified via BCA assay(Beyotime, Cat. No. P0010), separated by SDS-PAGE, and analyzed by Western blotting (ImageJ, 1.54g, Wayne Rasband, National Institutes of Health, Bethesda, MA, USA).

Data (mean ± SD, triplicate experiments) were analyzed in R (v4.2.2). Wilcoxon rank-sum test (Mann–Whitney U, non-parametric) was applied when normality was violated or sample sizes were small, including: (i) biomarker expression differences between disease and control groups across PMOS and MCI discovery/validation datasets; (ii) immune-signature comparisons; (iii) detection via Seurat FindMarkers (test.use = “wilcox”) for Scissor+ versus Scissor- clusters; and (iv) immune-cell subpopulation proportions. Moderated t-statistics from limma (empirical Bayes) were used for transcriptomic DEG calling and GSVA comparisons. Student’s *t*-test was adopted for two-group comparisons when data satisfied normality and homogeneity-of-variance assumptions. *p* < 0.05 was considered significant.

## 3. Results

### 3.1. Shared Mitochondria-Associated Transcriptional Signatures Were Identified in PMOS and MCI

The polyendocrine metabolic ovarian syndrome (PMOS) dataset contained 2960 DEGs (2824 upregulated and 136 downregulated). The mild cognitive impairment (MCI) dataset contained 2520 DEGs (973 upregulated and 1547 downregulated) (Figure 1A–D). The intersection of PMOS DEGs, MCI DEGs, and mitochondria-related genes (MRGs) yielded 16 mitochondria-associated genes with concordant expression (concordance relies on tissue transcriptomics; in vitro validation showed an exception for LYN in granulosa cells; see Section 3.7) (Figure 1E). These represent transcriptional signatures linking PMOS and MCI, not biomarkers validated in comorbidity cohort. Functional enrichment linked genes to protein autophosphorylation, mitochondrial inner membrane, mitochondrial intermembrane space, and TNF-activated receptor activity. KEGG analysis highlighted TNF signaling (*p* = 0.00805), NF-κB signaling (*p* = 0.00632), and mitophagy-related pathways (Figure 1F,G), suggesting that mitochondrial stress and inflammatory signaling converge. The DEG sensitivity framework, including FDR-ranked positions, relaxed effect size screening, and leave-one-dataset-out direction concordance for GSE34526 and GSE102293, is shown in Figure S1.

### 3.2. SCO2, LYN, and FGR Were Prioritized as Candidate Shared Biomarkers

Machine-learning-based feature prioritization uses shared mitochondrial-associated genes. All four algorithms (generalized linear model (GLM), random forest (RF), extreme gradient boosting (XGB), support vector machine (SVM)) were trained separately on both discovery datasets. Model performance was assessed using residuals and cross-validated AUC. The GLM delivered the best performance on the PMOS discovery dataset, whereas the random forest performed best on the MCI discovery dataset. Genes with an importance score > 20 were retained from each model, yielding 11 shared biomarkers across the PMOS and MCI groups (Figure 2A,B). Their expression was examined in the discovery/validation datasets. Among these, SCO2, LYN, and FGR showed consistent differential expression in the PMOS discovery cohort (*p* = 0.0229, 0.00787, 0.0115, respectively), MCI discovery dataset GSE63061 (*p* = 1.08 × 10^−5^, 4.49 × 10^−4^, 0.00140), and MCI validation dataset GSE63060 (*p* = 3.06 × 10^−6^, 3.42 × 10^−5^, 4.47 × 10^−5^) and were retained as biomarkers (Figure 2C,D). Because the discovery and validation datasets came from independent cohorts and tissue sources, these genes should be interpreted as putative cross-disease biomarkers rather than confirmed PMOS–MCI comorbidity markers. The feature-ranking reproducibility workflow, including repeated stratified cross-validation and resampling, is shown in Figure S2.

### 3.3. Exploratory Gene-Based Diagnostic Models Showed Potential but Require External Clinical Validation

Exploratory nomogram models were constructed for PMOS and MCI using SCO2, LYN, and FGR expression profiles (Figure 3A,C). The calibration curves suggested an agreement between the predicted and observed probabilities across the datasets (Figure 3B,D). These findings indicate that the three-gene panel may have potential for classifying PMOS and MCI. However, because no dataset contained clinically confirmed PMOS–MCI comorbidity and the PMOS datasets had limited sample sizes, these nomograms are purely exploratory computational tools and are not clinically validated diagnostic instruments for identifying PMOS-related cognitive impairment. The nomogram calibration details are provided in Figure S3.

### 3.4. Immune-Related Signatures Linked the Candidate Biomarkers to Inflammatory Dysregulation

Because the PMOS discovery datasets (GSE34526, GSE102293) use purified granulosa cells rather than immune-cell-rich tissue, CIBERSORT results reflect immune-related signatures, not infiltration. Estimates suggest changes in PMOS and MCI. In PMOS, monocyte (*p* = 0.0428) and CD8+ T-cell (*p* = 0.0287) signatures increased; resting CD4+ memory T-cell signatures decreased (*p* = 0.0110) (Figure 4A,B). In MCI, memory B-cell (*p* = 0.0189) and resting NK-cell (*p* = 0.0374) signatures increased; activated NK-cell (*p* = 0.0459) and resting CD4+ memory T-cell signatures decreased (*p* = 0.0128) (Figure 4C,D). Analysis showed that SCO2, LYN, and FGR correlated positively with CD8+ T-cell signatures in PMOS and negatively with resting CD4+ memory T-cell signatures in both. The strongest correlations occurred in PMOS, where FGR, LYN, and SCO2 correlated with resting CD4+ memory T cells (r = −0.99, −0.95, −0.93). In MCI, correlations were weaker but consistent (r = −0.28, −0.27, −0.27). SCO2 positively correlated with memory B-cell signatures in MCI (r = 0.22) (Figure 4E,F). The findings support an association between biomarkers and immune-inflammatory remodeling, although PMOS estimates require caution given the granulosa cell source. Sensitivity analysis comparing CIBERSORT with ssGSEA or xCell scores is summarized in Figure S4.

### 3.5. Pathway-Level Analyses Supported an Immune-Inflammatory and Mitochondrial Stress Axis

Gene set enrichment analysis (GSEA) and gene set variation analysis (GSVA) characterized the associations between SCO2, LYN, and FGR. In PMOS, SCO2 was linked to KEGG_LEISHMANIA_INFECTION and KEGG_ALLOGRAFT_REJECTION (Figure 5A), LYN was linked to KEGG_LEISHMANIA_INFECTION and KEGG_HEMATOPOIETIC_CELL_LINEAGE (Figure 5B), and FGR was linked to KEGG_LEISHMANIA_INFECTION and KEGG_FC_GAMMA_R_MEDIATED_PHAGOCYTOSIS (Figure 5C). In MCI, SCO2 was linked to KEGG_LYSOSOME (Figure 5D), LYN to KEGG_FC_GAMMA_R_MEDIATED_PHAGOCYTOSIS (Figure 5E), and FGR to KEGG_CHEMOKINE_SIGNALING_PATHWAY (Figure 5F). Co-enrichment analysis identified 56 overlapping pathways for FGR, 41 for LYN, and 39 for SCO2 in both PMOS and MCI (Figure 5G–I). Disease–gene pathway networks were observed for FGR (Figure 5J), LYN (Figure 5K), and SCO2 (Figure 5L). Network visualization suggested that these genes are linked to inflammatory pathways, including the JAK-STAT and MAPK signaling pathways. GSVA identified 53 enriched pathways in PMOS and 94 in MCI, with 27 shared pathways, including the Toll-like receptor and Notch signaling pathways (Figure 6). These findings support a model in which mitochondrial dysfunction, innate immune activation, and inflammatory kinase signaling converge in both PMOS and MCI. Predicted regulatory, disease–gene, drug–target, and molecular docking evidence are shown in Figure S5 and Table S4. These in silico analyses are hypothesis-generating and not target-validated.

### 3.6. Single-Cell Analysis Localized Disease-Associated Signals to Fibroblast and Immune-Cell States in PMOS Ovarian Tissue

Single-cell RNA sequencing (scRNA-seq) of PMOS ovarian tissue identified 12 clusters across five populations: immune, fibroblast, secretory epithelial, dendritic, and theca cells (Figure 7A–E). The quality control metrics are shown in Figure S6. Scissor analysis linked single-cell profiles to bulk PMOS features (Figure S7), identifying fibroblasts as the predominant Scissor+ population (PMOS-associated) and immune cells as the major Scissor− population (control-associated) in the PMOS group. Unclassified Scissor0 cells showed no significant phenotypic associations (Figure 7F,G). Differential expression between Scissor+ and Scissor- cells identified 87 genes enriched in xenobiotic stimulus, protein phosphatase regulator activity, adherens junctions, and HIF-1 signaling (Figure S1B,C). Cell–cell communication analysis revealed weaker interactions between fibroblasts and immune cells in Scissor+ cells, whereas dendritic and secretory epithelial cells showed stronger interactions (Figure 8A–D). Immune cells were selected for trajectory analysis. Pseudotime analysis showed SCO2 and LYN expression in the early and late states, respectively, whereas FGR was undetectable (Figure 9A). The absence of FGR may reflect low expression or tissue-specific patterns, which limits cellular context validation within this framework. Secondary clustering identified plasmacytoid dendritic cells, classical monocytes, and pro-B cells; classical monocytes were most abundant in disease samples (40.5%), while pro-B cells dominated the controls (47.5%) (Figure 9B–E).

### 3.7. Cell-Model Expression Assessment

Expression validation was carried out using ovarian granulosa and microglial inflammatory injury models. In granulosa-cell comparisons versus KGN cells, SCO2 and FGR showed significantly elevated mRNA and protein levels in COV434 cells, whereas LYN mRNA was significantly decreased (all *p* < 0.05) (Figure 10A–E). In lipopolysaccharide (LPS)-treated BV2 microglial cells, SCO2, LYN, and FGR mRNA and protein levels increased versus control BV2 cells (all *p* < 0.05) (Figure 10F–K). These results are largely consistent with the predicted upregulation, with the notable exception of LYN in granulosa-cell lines, and support candidate-gene expression relevance; therefore, further functional perturbation experiments are required to define causal roles in mitochondrial stress and inflammatory signaling. Cell-line quality-control and raw-data reporting, including STR authentication, mycoplasma testing, passage range, quantitative real-time polymerase chain reaction (qRT-PCR) Ct-values, primer efficiency, and uncropped Western blot membranes, are provided in Figure S8.

## 4. Discussion

### 4.1. Principal Findings

We performed an integrative transcriptomic analysis to identify mitochondria-associated immune-inflammatory signatures across the PMOS and MCI datasets. Intersecting PMOS- and MCI-related DEGs with MRGs, followed by feature ranking and cross-dataset assessment, prioritized SCO2, LYN, and FGR as candidate genes. Pathway analyses indicated inflammatory and mitochondrial stress-related pathways, including TNF, NF-κB, mitophagy-related processes, Fcγ receptor-mediated phagocytosis, chemokines, JAK-STAT, MAPK, Toll-like receptor, and Notch signaling, suggesting convergent mitochondrial stress and immune-inflammatory remodeling across PMOS and MCI contexts.

Interpreting the findings within this design is challenging. The PMOS and MCI datasets were obtained from independent cohorts and tissues and did not confirm the diagnosis of PMOS–MCI. However, these genes have not been validated as biomarkers for specific comorbidities. Instead, SCO2, LYN, and FGR were prioritized as mitochondria-associated immune-inflammatory signatures based on cross-dataset bioinformatic evidence. This study generated mechanistic hypotheses and a prioritization framework for clinical/functional validation but not definitive causal evidence or a clinically applicable diagnostic model.

### 4.2. Mitochondrial Stress and Immune-Inflammatory Remodeling as a Candidate Shared Axis

Mitochondrial dysfunction has been implicated in PMOS and neurocognitive disorders. In PMOS, mitochondrial abnormalities may contribute to impaired oxidative phosphorylation (OXPHOS), reactive oxygen species (ROS) production, insulin resistance, and inflammation. Mitochondrial dysfunction, oxidative stress, neuroinflammation, and metabolic alterations mark MCI progression and neurodegenerative conditions [36]. This study suggests that mitochondrial transcriptional changes may converge with immune-inflammatory signaling in the PMOS and MCI datasets.

This overlap does not imply shared mechanisms. Compartments may reflect immunometabolic disturbances. Granulosa cells mediate endocrine–metabolic regulation, and dysfunction may contribute to PCOM-linked disturbances in PMOS [7,8,21], whereas blood transcriptomes may capture immune-inflammatory alterations linked to neurocognitive vulnerability. Cross-tissue comparisons identify pathway convergence, not tissue-specific biomarkers. We integrated two-tissue profiles as a filter, not to assess similarity. Expression shifts reflect conserved pathways, including SCO2, LYN, FGR. PMOS is a systemic endocrine–metabolic–immune syndrome affecting organs [37]. Parallel ovarian and immune-cell abnormalities may reflect shared pathology [21,38,39].

NF-κB, TNF, JAK-STAT, and MAPK are inflammatory cascades. SCO2, LYN, and FGR are PMOS–MCI signatures rather than systemic-inflammation markers because, unlike pro-inflammatory cytokines, they have mitochondrial functions: SCO2 supports cytochrome c oxidase assembly for respiration [40]; LYN and FGR, SRC-family kinases, are in MitoCarta 3.0 with mitochondrial relevance [41]. They reflect mitochondrial–immunometabolism perturbation, are upregulated in PMOS granulosa cells and MCI blood, prioritized by GLM and RF among 16 mitochondrial-related genes, and dysregulated in single-cell myeloid and B-lymphoid subpopulations. These signatures require testing in inflammation.

### 4.3. SCO2 as a Mitochondrial Respiratory Stress-Related Candidate Signature

SCO2, linked to mitochondria, encodes protein essential for cytochrome c oxidase assembly and IV function [42,43]. Dysregulation of cytochrome c oxidase assembly impairs oxidative phosphorylation and energy metabolism. In metabolic diseases, mitochondrial dysfunction may aggravate insulin resistance, oxidative stress, and inflammation [44,45]. Increased SCO2 expression in PMOS and MCI datasets may indicate compensatory/stress-responsive respiratory imbalance.

NF-κB regulates mitochondrial respiration via SCO2-related pathways [46], linking inflammation to mitochondrial adaptation. Enrichment highlighted NF-κB and TNF pathways, suggesting SCO2 involvement in inflammation-associated mitochondrial stress. Mitochondrial dysfunction impairs synaptic plasticity, energy homeostasis, and cognition, contributing to MCI and Alzheimer’s vulnerability [47]. SCO2 may be a mitochondrial component of the PMOS–MCI transcriptional signature. Data do not establish that SCO2 mediates cognitive impairment in PMOS. Transcriptomic and pathway analyses linked SCO2 to inflammatory and mitochondrial pathways, making it a mechanistic candidate. Experiments assessing oxidative phosphorylation, ATP production, membrane potential, reactive oxygen species, and inflammatory cytokines after SCO2 perturbation are required to determine its role in CRC.

### 4.4. LYN/FGR as Immune-Inflammatory Kinase-Related Candidate Signatures

LYN and FGR are members of the SRC kinase-related signaling network involved in immune regulation, inflammatory activation, and intracellular signal transduction. Although included in this study’s mitochondria-related gene set, their relevance lies in mitochondria-associated immune-inflammatory signaling rather than in direct mitochondrial function. This distinction avoids overinterpretation of classical mitochondrial gene roles.

LYN mediates immune, inflammatory, and metabolic signaling. SRC kinase LYN modulates activation and receptor transduction [48]. Studies have linked LYN to PI3K/AKT signaling and insulin resistance, which are central to PMOS pathophysiology [49,50]. LYN is associated with microglial activation, neuroinflammation, amyloid-β phagocytosis, neuronal damage, and survival in Alzheimer’s-related models [51,52]. These observations support LYN as an immune-inflammatory node linking metabolic dysfunction and neuroinflammatory vulnerability. LYN was upregulated in PMOS and MCI datasets and linked to immune-related patterns. Single-cell analysis revealed LYN in immune cell trajectories, supporting its relevance in PMOS tissue. Evidence for LYN remains cross-dataset- and context-dependent and has not been validated.

FGR, an SRC kinase studied in PMOS and MCI, is involved in inflammatory signaling, Fcγ receptor-mediated activation, phagocytosis, oxidative stress, and cytokine regulation [53,54]. FGR inhibition attenuates mitochondrial dysfunction, oxidative stress, and neuroinflammation in sepsis-associated encephalopathy models, suggesting an immune activation–mitochondrial injury link [55,56]. Studies have supported the role of mitochondrial quality control, kinase signaling, and neuroinflammatory regulation in neurological disorders [57,58].

FGR and LYN were upregulated in bulk datasets, but FGR was not detected in the PMOS single-cell pseudotime trajectory. FGR’s nomination relies on bulk evidence: differential expression in discovery and validation datasets, machine-learning prioritization, and molecular-docking results (−6.7 kcal/mol). Single-cell pseudotime provides mechanistic insight, not a core biomarker-selection criterion. Non-detection does not exclude utility, because genes may show disease-related expression shifts without trajectory-dependent fluctuations. Possible reasons include 10 × Genomics dropout effects [59] and ovarian versus peripheral-blood immune-cell differences affecting FGR expression [60]. Functionally, SCO2 and LYN have broader basal roles, whereas FGR depends on extrinsic stimulation [40,61]. Thus, FGR remains a plausible bulk-derived candidate despite limited ovarian single-cell detectability.

### 4.5. Cell-Model Expression Assessment Provided Preliminary Experimental Support

SCO2, LYN, and FGR were upregulated in PMOS and MCI, but LYN was downregulated in COV434 versus KGN lines, whereas SCO2 and FGR were elevated at both levels. In LPS-stimulated BV2, three increased, likely because COV434/KGN lines do not reconstruct the microenvironment. LYN, an SRC-family kinase, may reflect transformation and extrinsic cues [41]. Discordance does not negate LYN’s candidacy but requires validation in primary granulosa cells.

The LPS-treated BV2 microglial model is an endotoxin-driven neuroinflammatory surrogate and does not recapitulate PMOS-associated neuroinflammation. PMOS-related cognitive impairment arises from chronic endocrine–metabolic disturbances, including hyperandrogenism and insulin resistance-related circulating mediators, not bacterial-derived LPS [62]. This model evaluates the expression under neuroinflammatory stress but cannot reconstruct the hormonal–metabolic microenvironment driving PMOS-linked alterations. This limitation warrants future in vitro androgen-or insulin resistance-conditioned microglial co-culture studies to mimic PMOS-originated neuroinflammation.

### 4.6. Cellular Context Evidence from PMOS Single-Cell RNA-Seq

Single-cell RNA sequencing (scRNA-seq) aided bulk transcriptomic interpretation. The PMOS dataset revealed immune cells, fibroblasts, secretory epithelial cells, dendritic cells, and theca cells. Scissor analysis linked PMOS patterns to states and suggested that fibroblasts were predominant scissor-positive cells, whereas immune cells were scissor-negative, indicating that stromal and immune components may contribute to PMOS-associated microenvironmental remodeling.

Although fibroblasts were the predominant Scissor-positive population, immune cells were selected for trajectory analysis because the hypothesis centered on immune-inflammatory mitochondrial signatures, SCO2, and LYN expression. Annotation identified plasmacytoid dendritic cells, monocytes, and pro-B cells; monocytes predominated in diseased samples, but pro-B cells in controls, supporting immune-inflammatory remodeling and innate immune activation in PMOS-related microenvironmental changes. NF-κB activation is implicated in inflammation and insulin resistance in PMOS [63,64], and NF-κB-related pathways contribute to dysfunction and cognitive impairment in neurological models [65,66,67]. Shared NF-κB, TNF-, Toll-like receptor, and kinase pathways suggest an overlap between PMOS and MCI, favoring SCO2 and LYN over FGR.

### 4.7. Interpretation of Immune-Related Deconvolution Results

Deconvolution suggests remodeling in PMOS and MCI. In PMOS, monocyte-like and CD8+ T-cell signatures increased, whereas resting CD4+ memory T-cell signatures decreased. In MCI, memory B-cell and resting natural killer signatures increased, whereas activated natural killer and resting CD4+ memory T-cell signatures decreased. SCO2, LYN, and FGR were negatively correlated with resting CD4+ memory T-cell signals, strongest in PMOS (r = −0.99, −0.95, and −0.93 for FGR, LYN, and SCO2, respectively) and weaker in MCI (r = −0.28, −0.27, and −0.27, respectively).

The findings warrant caution because Cell-type Identification by Estimating Relative Subsets of RNA Transcripts (CIBERSORT) LM22 was developed for immune mixtures. In granulosa data, it does not measure immune abundance. CIBERSORT-derived outputs are exploratory, not decisive evidence for immune microenvironment. Conclusions for SCO2, LYN, and FGR are supported by expression across datasets, machine-learning prioritization, and single-cell RNA-seq observations. No orthogonal immune datasets are available for validation. Deconvolution and enrichment support immune-inflammatory remodeling relevant to the PMOS–MCI signature. Immune- and oxidative stress-related alterations occur in inflammatory and metabolic disorders, including diabetic retinopathy and ovulation dysfunction conditions [68,69], supporting plausibility.

### 4.8. Translational Implications

SCO2, LYN, and FGR frames to study immune-mitochondrial links between PMOS and MCI were identified. Unlike studies on mitochondrial dysfunction in PMOS or neurodegenerative diseases [38,39], cross-dataset analysis integrated reproductive, metabolic, immune, and neuroinflammatory contexts, prioritizing genes for clinically characterized PMOS–MCI cohorts.

Translationally, this study prioritizes the candidates. The models suggested that SCO2, LYN, and FGR could classify diseases in the PMOS and MCI datasets. However, developed from independent datasets rather than a PMOS–MCI comorbid cohort, they were not validated. Their utility is a hypothesis-generating panel for validation in cohorts with standardized PMOS diagnoses, cognitive assessments, metabolic profiling, inflammatory markers, and longitudinal follow-up. SCO2 reflects mitochondrial respiratory stress, whereas LYN and FGR represent immune-inflammatory kinase signaling linked to inflammatory amplification and mitochondrial injury, respectively. The therapeutic implications remain preliminary, and the targets require testing.

### 4.9. Strengths and Limitations

The strengths of this study included the use of PMOS and MCI datasets focused on mitochondria-associated genes, thereby reducing overlap; prioritizing genes with feature-ranking algorithms and assessing them in discovery and validation datasets; and integrating analyses, immune-related deconvolution, PMOS single-cell RNA-seq contextualization, and model assessment, thereby strengthening the evidence base and providing a valid framework.

The PMOS cohort (*n* = 16) limited the detection of differential expression and stability. Low power may miss DEGs, unstable variance estimation yields false positives, and cohort-estimated priors limit limma’s empirical Bayes variance shrinking. GLM may overfit noise. Stratified 10-fold cross-validation yielded 1–2 folds, AUC, and unstable coefficient values. We used elastic net regression (alpha = 0.2) with L2-penalization and multi-algorithm filtering/validation. GLM was checked against random forest, SVM, and XGBoost; genes changed in PMOS (GSE137684) and MCI (GSE63060/GSE63061). We lacked a PMOS–MCI validation cohort; therefore, we could not assess the three-gene signature.

Immune-related deconvolution reflects transcriptional, not immune, signatures. FGR was not detected in the PMOS pseudotime trajectory. In vitro experiments verified SCO2, LYN, and FGR expression, but not their mechanisms. Findings rely on multi-omics inference and validation. No loss- or gain-of-function assays assessed mitochondrial stress or immune-inflammatory signaling; however, such assays are needed. PMOS DEG screening with (*n* = 16) precluded FDR-level discovery; sensitivity analyses (Figure S1) and MRG intersection mitigated this, but it remains exploratory.

### 4.10. Future Directions

To detect shared PMOS–MCI signatures, larger granulosa-cell and peripheral-blood cohorts will permit FDR-controlled differential expression testing. Meta-analysis across PMOS granulosa datasets can boost power before cross-disease intersection. Validating candidates in multi-omics datasets and PMOS–MCI comorbidity cohorts, alongside permutation-based evaluation, will exclude chance-driven false-positive overlaps.

To validate the three-gene panel, a multicenter nested case–control design is ideal. Women with PMOS aged 35–50 are followed for 5–10 years with cognitive assessments. At endpoint, participants are stratified into PMOS–MCI cases and PMOS controls. PBMC mRNA levels of SCO2, LYN, and FGR are quantified, and performance is assessed using AUC, calibration curves, and decision-curve analysis (DCA). Mechanistic validation includes siRNA-mediated knockdown and CRISPR-Cas9 knockout of SCO2, LYN, and FGR in granulosa and microglial models. After perturbation, mitochondrial phenotypes—oxidative phosphorylation, ATP yield, membrane potential, and reactive oxygen species—and inflammatory outputs, including cytokine release and NF-κB/TNF signaling, are measured. Overexpression assays will provide cross-validation. These assays will define the genes’ relationships to mitochondrial–immune dysregulation in PMOS–MCI.

Static cultures cannot recapitulate multi-organ/tissue–brain crosstalk or endocrine–metabolic mediators underlying PMOS-associated MCI. In vivo studies need chronic androgen-induced PMOS models with hyperandrogenism, insulin resistance, ovarian dysfunction, and low-grade inflammation. Novel-object recognition/Y-maze can assess impairment. Multi-tissue sampling permits SCO2, LYN, and FGR profiling of immunometabolic cross-talk. Tissue-specific knockout/overexpression can dissect whether reproductive/immune-compartment perturbations drive molecular/behavioral changes.

## 5. Conclusions

Transcriptomic analysis identified SCO2, LYN, and FGR as correlative mitochondria-associated immune-inflammatory signatures for polyendocrine metabolic ovarian syndrome (PMOS) and mild cognitive impairment (MCI) via immunometabolic and inflammatory pathways. SCO2 was associated with respiratory and oxidative-stress-related changes, whereas LYN and FGR were associated with immune activation and SRC-family-kinase-related signaling within the NF-κB/TNF-axis. These genes correlate with immune dysregulation and metabolic-stress-related transcriptional profiles linked to PMOS-relevant cognitive-impairment-associated features. Notably, these cross-dataset findings are correlative and do not establish causality or validate comorbidity-specific biomarkers. Gene-based diagnostic models were constructed for PMOS and MCI. Validation in PMOS–MCI comorbidity cohorts plus functional-intervention experiments remains essential before inference or translational applications.

## Figures and Tables

**Figure 1:**
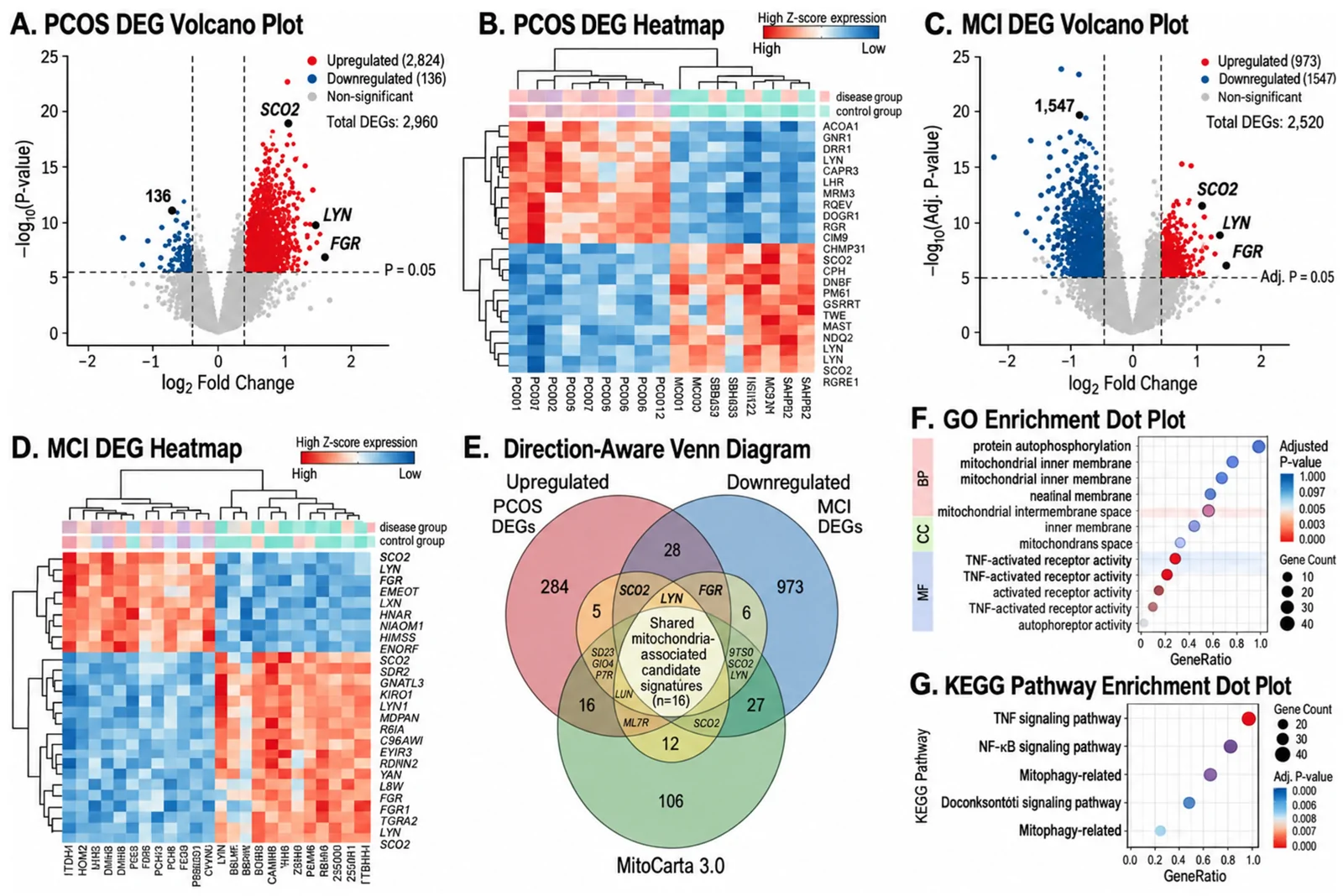
Shared mitochondria-associated transcriptional signatures identified from polyendocrine metabolic ovarian syndrome (PMOS) and mild cognitive impairment (MCI) transcriptomic datasets. (**A**) PMOS differentially expressed gene (DEG) volcano plot; (**B**) PMOS DEG heatmap; (**C**) mild cognitive impairment (MCI) DEG volcano plot; (**D**) MCI DEG heatmap; (**E**) direction-aware Venn intersection of PMOS DEGs, MCI DEGs, and mitochondria-related genes (MRGs) from MitoCarta 3.0; (**F**) Gene Ontology (GO) enrichment of shared mitochondria-associated genes; (**G**) Kyoto Encyclopedia of Genes and Genomes (KEGG) enrichment of shared mitochondria-associated genes.

**Figure 2:**
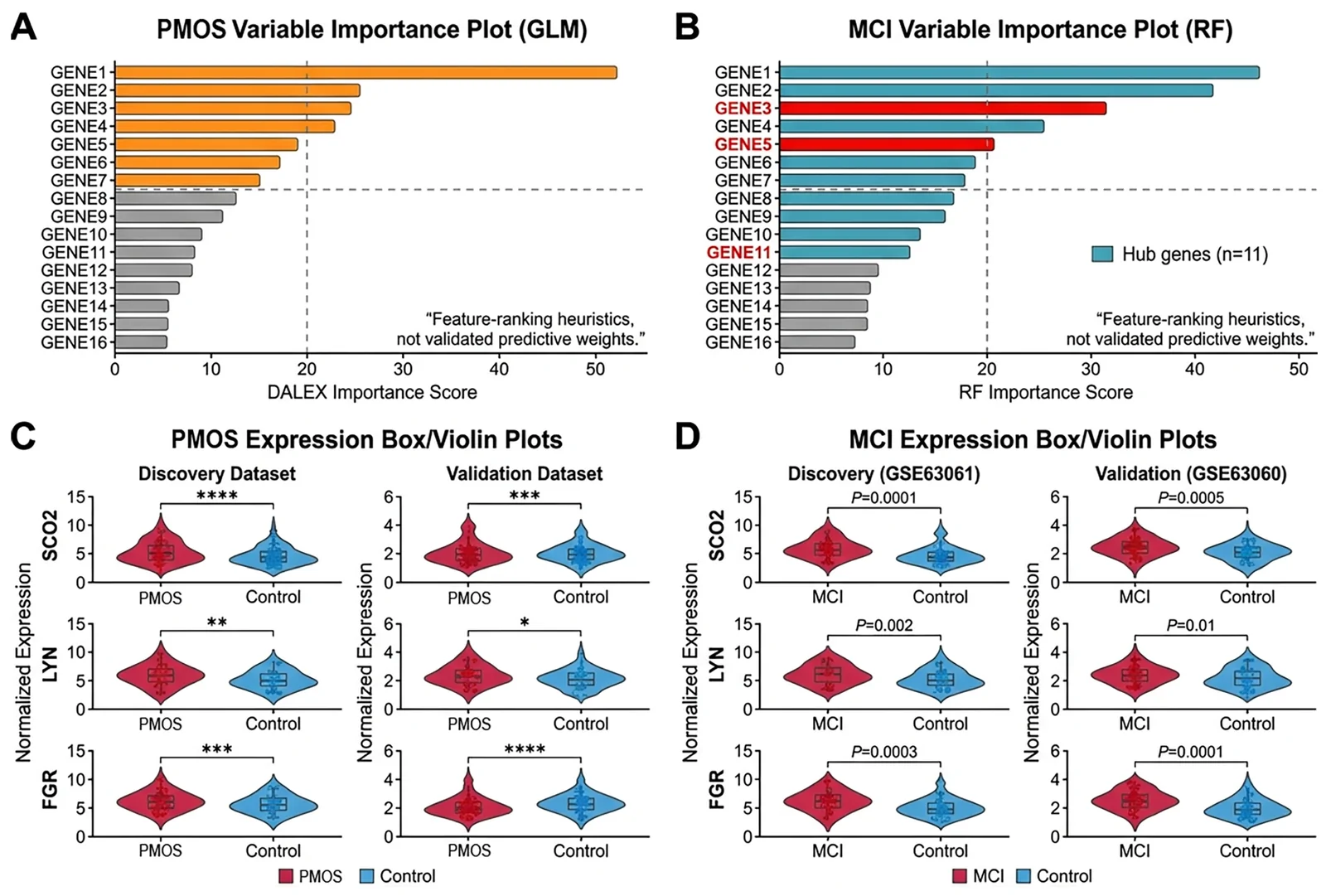
Machine learning feature prioritization and cross-dataset expression validation of candidate signatures. (**A**) Polyendocrine metabolic ovarian syndrome (PMOS) feature-importance ranking; (**B**) mild cognitive impairment (MCI) feature-importance ranking; (**C**) SCO2, LYN, and FGR expression in PMOS discovery and validation datasets; (**D**) SCO2, LYN, and FGR expression in MCI discovery and validation datasets. * *p* < 0.05, ** *p* < 0.01, *** *p* < 0.001, **** *p* < 0.0001.

**Figure 3:**
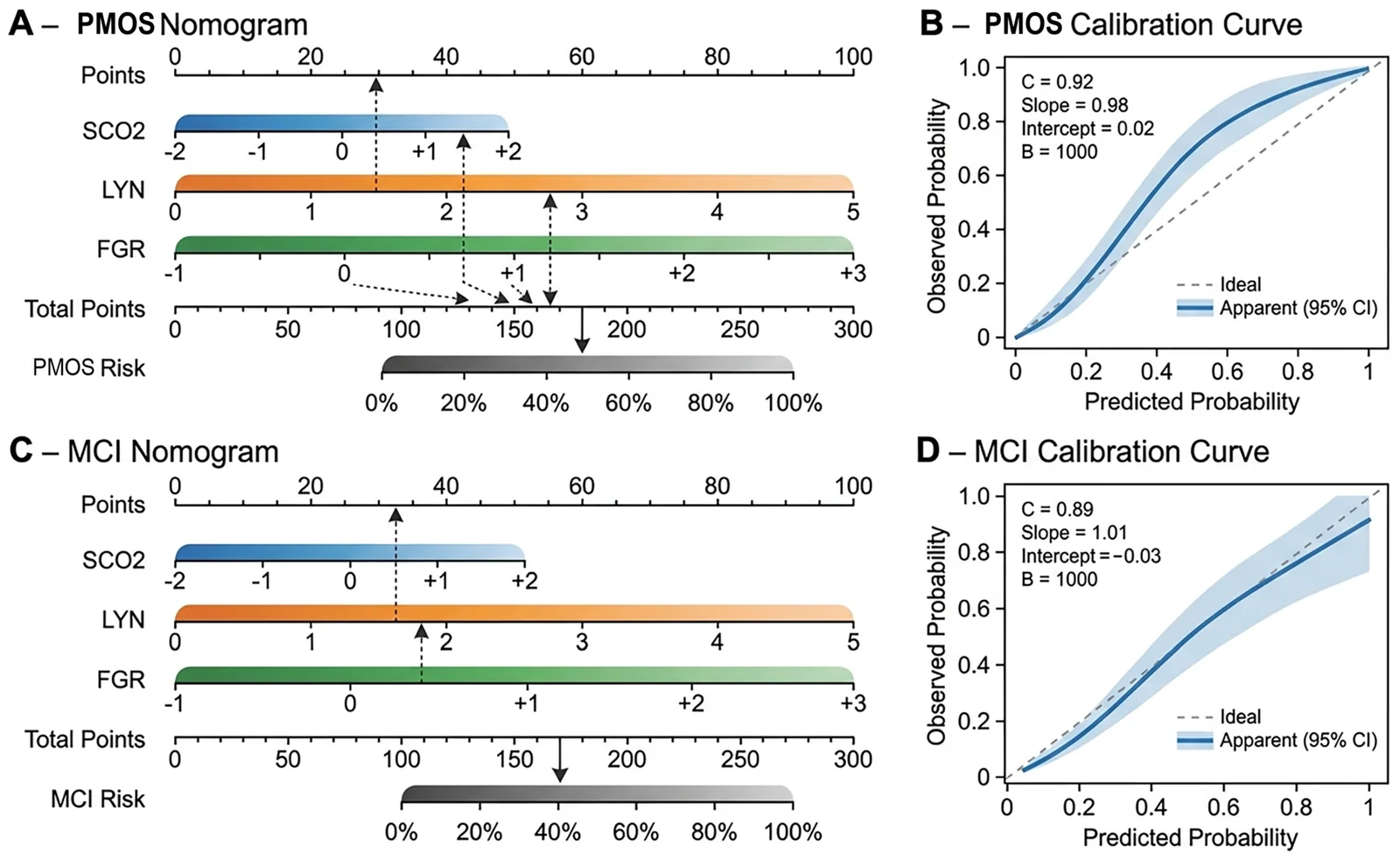
Exploratory gene-based nomogram classification models built using SCO2, LYN, and FGR expression profiles. (**A**) Polyendocrine metabolic ovarian syndrome (PMOS) nomogram based on SCO2, LYN, and FGR; (**B**) PMOS calibration curve assessing the agreement between predicted and observed probabilities; (**C**) mild cognitive impairment (MCI) nomogram based on SCO2, LYN, and FGR; (**D**) MCI calibration curve. In A and C, black arrows and dashed projection lines illustrate how SCO2, LYN, and FGR values are converted to points, summed as total points, and translated into the predicted PMOS or MCI risk.

**Figure 4:**
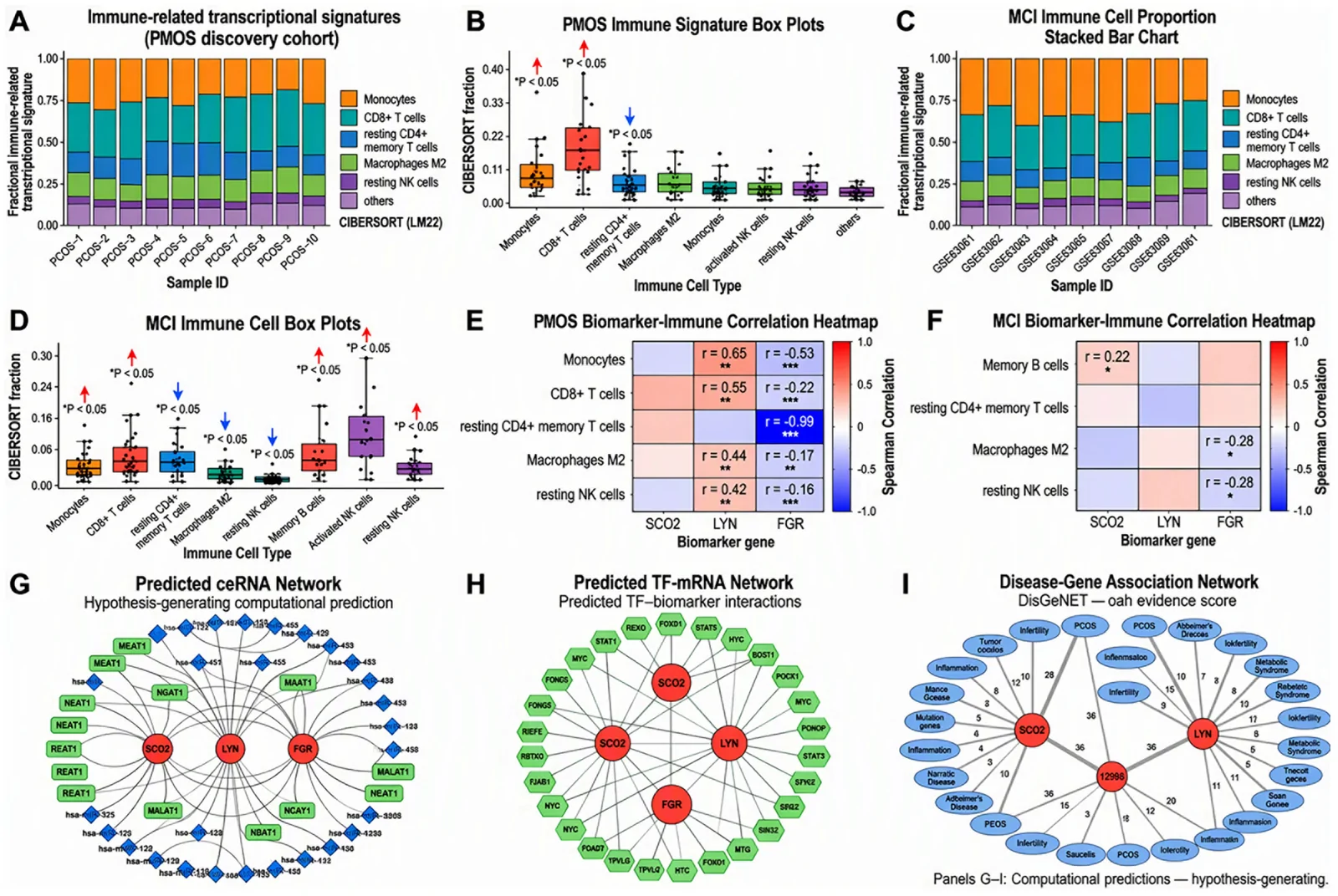
Immune-related transcriptional signature analysis and in silico regulatory network prediction. (**A**) Polyendocrine metabolic ovarian syndrome (PMOS) immune-related transcriptional signature distribution; (**B**) differential PMOS immune-related signatures; (**C**) mild cognitive impairment (MCI) immune-cell estimate distribution; (**D**) differential MCI immune-cell estimates; (**E**) PMOS biomarker–immune correlation heatmap; (**F**) MCI biomarker–immune correlation heatmap; (**G**) predicted competing endogenous RNA (ceRNA) network; (**H**) predicted transcription factor (TF)–mRNA network; (**I**) disease–biomarker association network. Red upward arrows and blue downward arrows in B and D indicate immune-related signatures that were significantly higher or lower, respectively, in the PMOS or MCI group than in the corresponding control group (*p* < 0.05). * *p* < 0.05, ** *p* < 0.01, *** *p* < 0.001.

**Figure 5:**
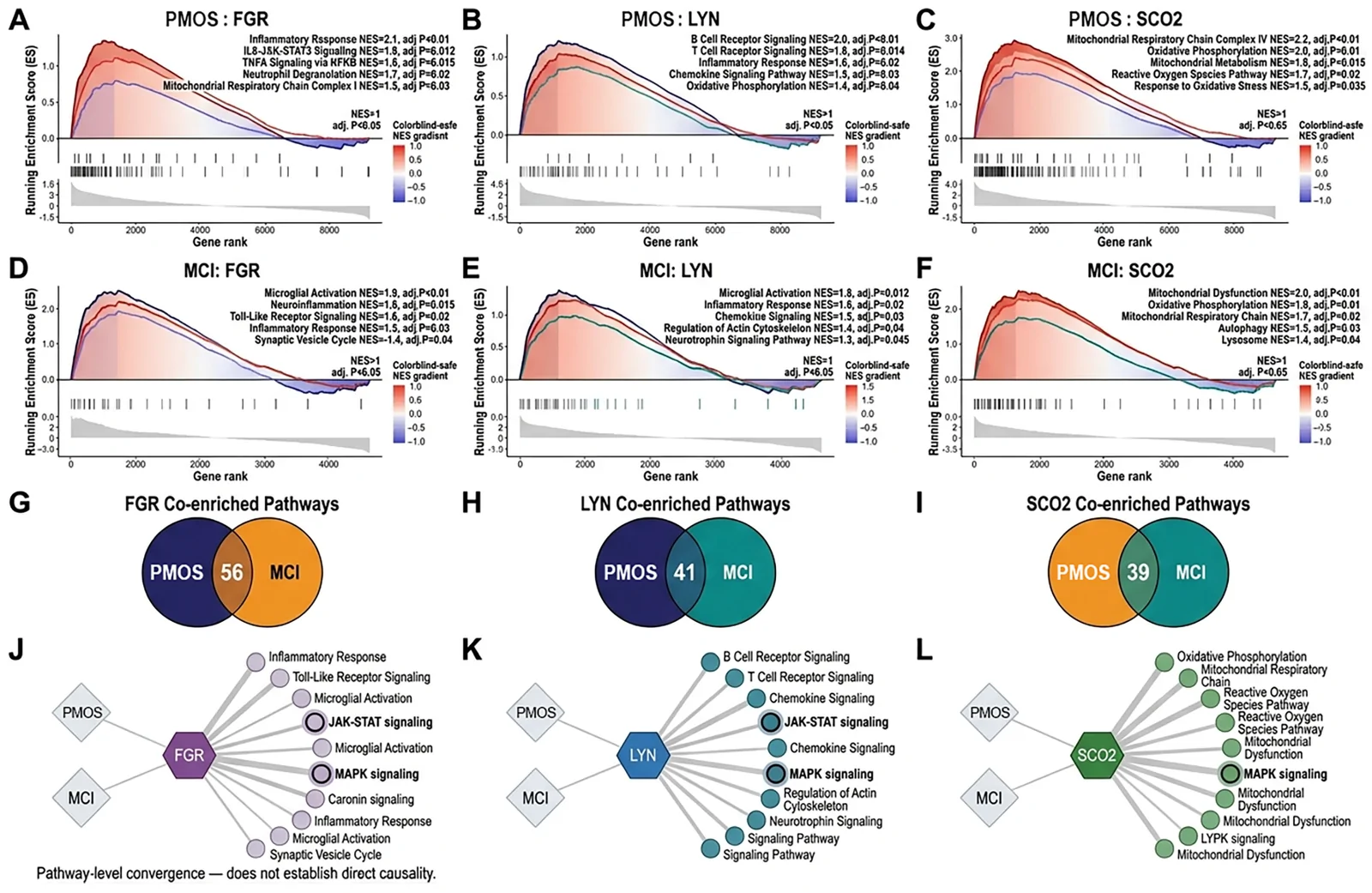
Gene set enrichment analysis (GSEA) revealing pathways associated with SCO2, LYN, and FGR in the PMOS and MCI datasets. Analyses identified overlapping immune-inflammatory and mitochondrial stress-related pathways across the two diseases. (**A**–**C**) Polyendocrine metabolic ovarian syndrome (PMOS) GSEA plots for SCO2, LYN, and FGR; (**D**–**F**) mild cognitive impairment (MCI) GSEA plots for SCO2, LYN, and FGR; (**G**–**I**) PMOS–MCI shared pathway Venn diagrams for FGR, LYN, and SCO2; (**J**–**L**) disease–gene-pathway networks for FGR, LYN, and SCO2.

**Figure 6:**
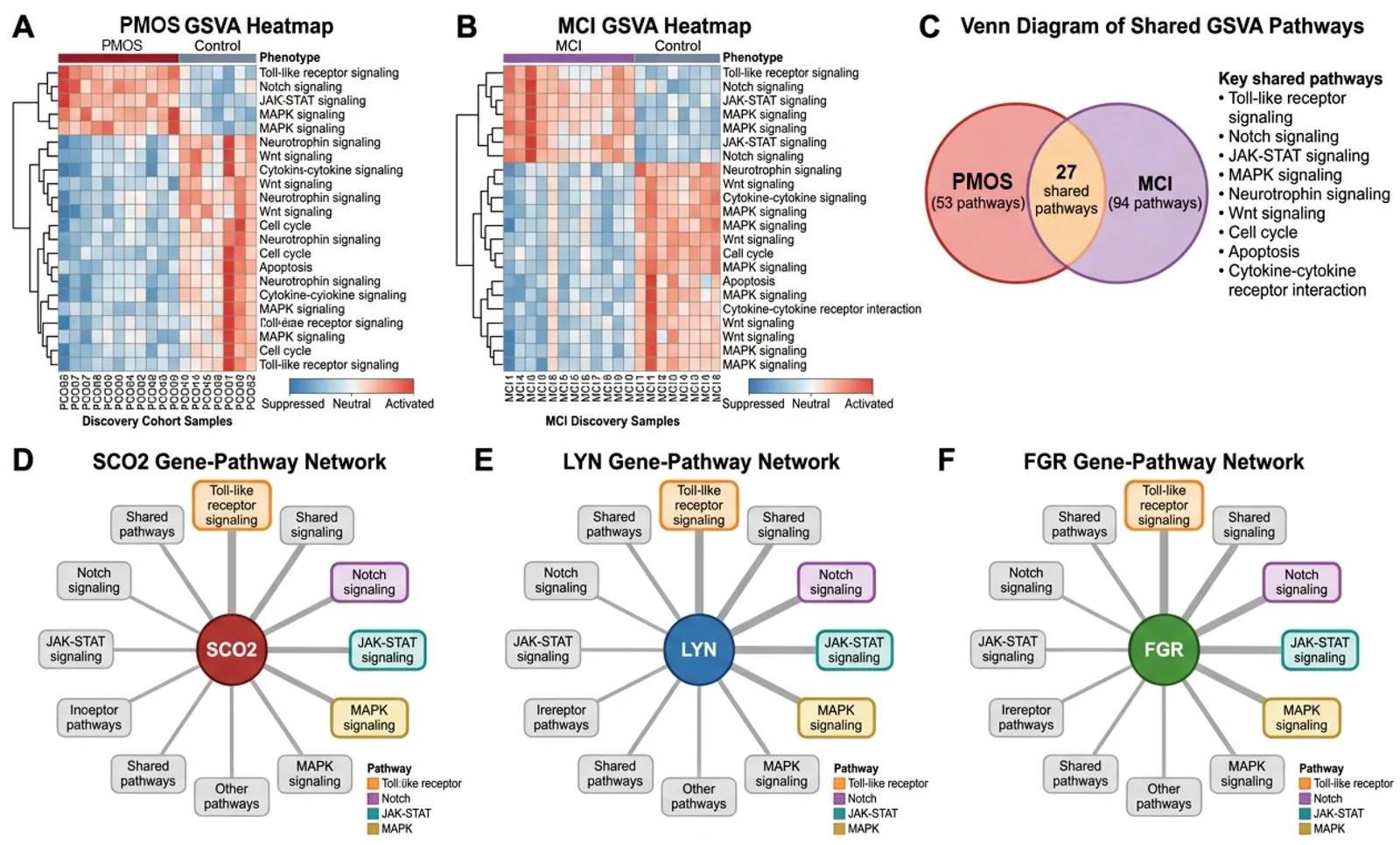
Gene set variation analysis (GSVA) for pathway-level convergence between polyendocrine metabolic ovarian syndrome (PMOS) and mild cognitive impairment (MCI). GSVA scores were used to compare pathway activity profiles between disease and control groups. (**A**) PMOS GSVA pathway heatmap; (**B**) MCI GSVA pathway heatmap; (**C**) shared PMOS–MCI GSVA pathway Venn diagram; (**D**–**F**) SCO2-, LYN-, and FGR-linked shared pathway networks.

**Figure 7:**
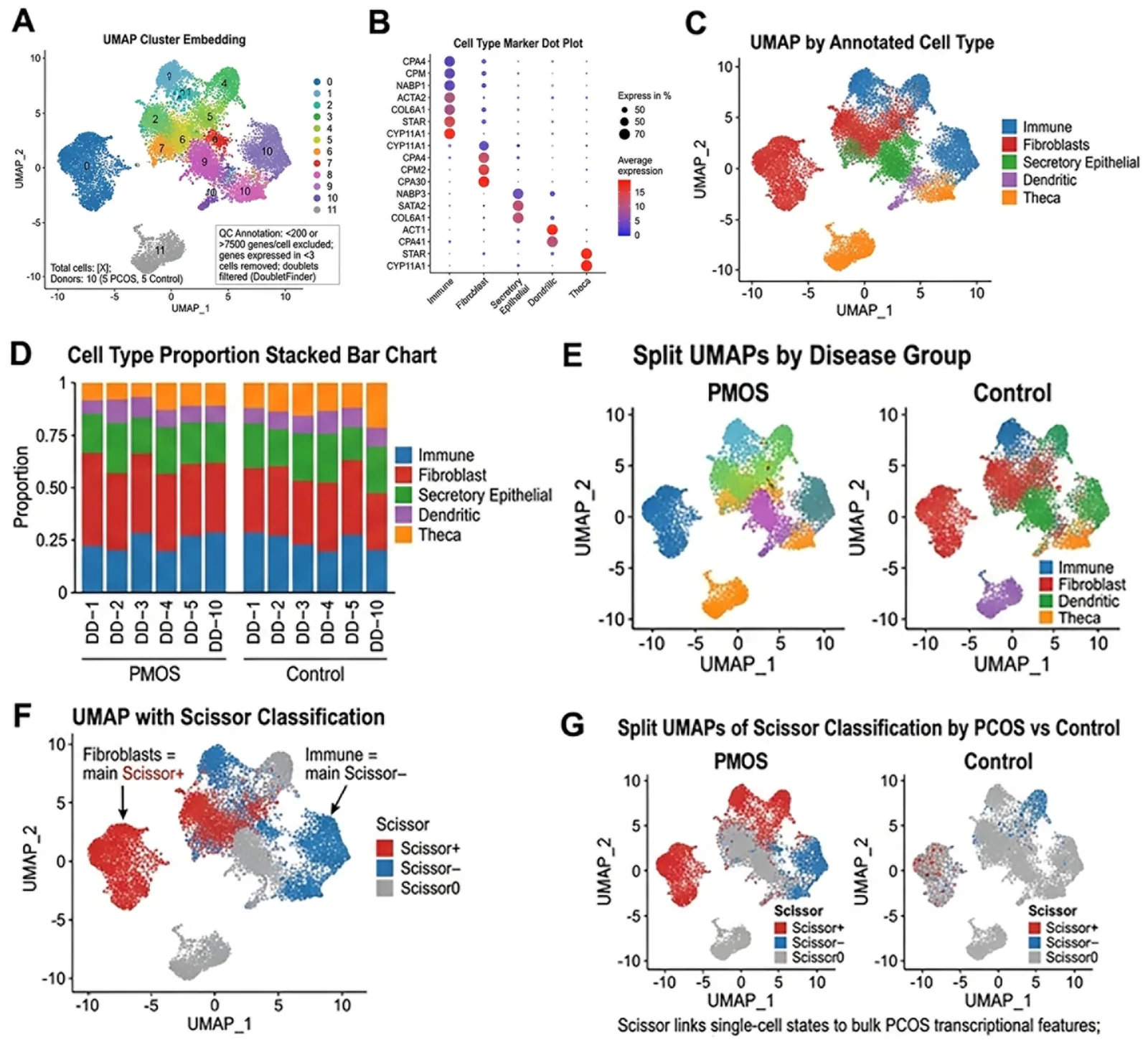
Single-cell RNA-sequencing (scRNA-seq) profiling of polyendocrine metabolic ovarian syndrome (PMOS) ovarian tissue combined with Scissor analysis to link single-cell states to bulk PMOS transcriptomic signatures. (**A**) Uniform Manifold Approximation and Projection (UMAP) of unsupervised cell clusters; (**B**) cell-type marker dot plot; (**C**) UMAP of annotated cell types; (**D**) cell-type proportions by group; (**E**) split UMAPs for PMOS and control samples; (**F**) Scissor-classified UMAP showing bulk-phenotype-associated cell states; (**G**) distribution of Scissor-defined cell states. Scissor+ denotes PMOS-associated cells; Scissor- denotes control-associated cells; Scissor0 denotes non-associated cells.

**Figure 8:**
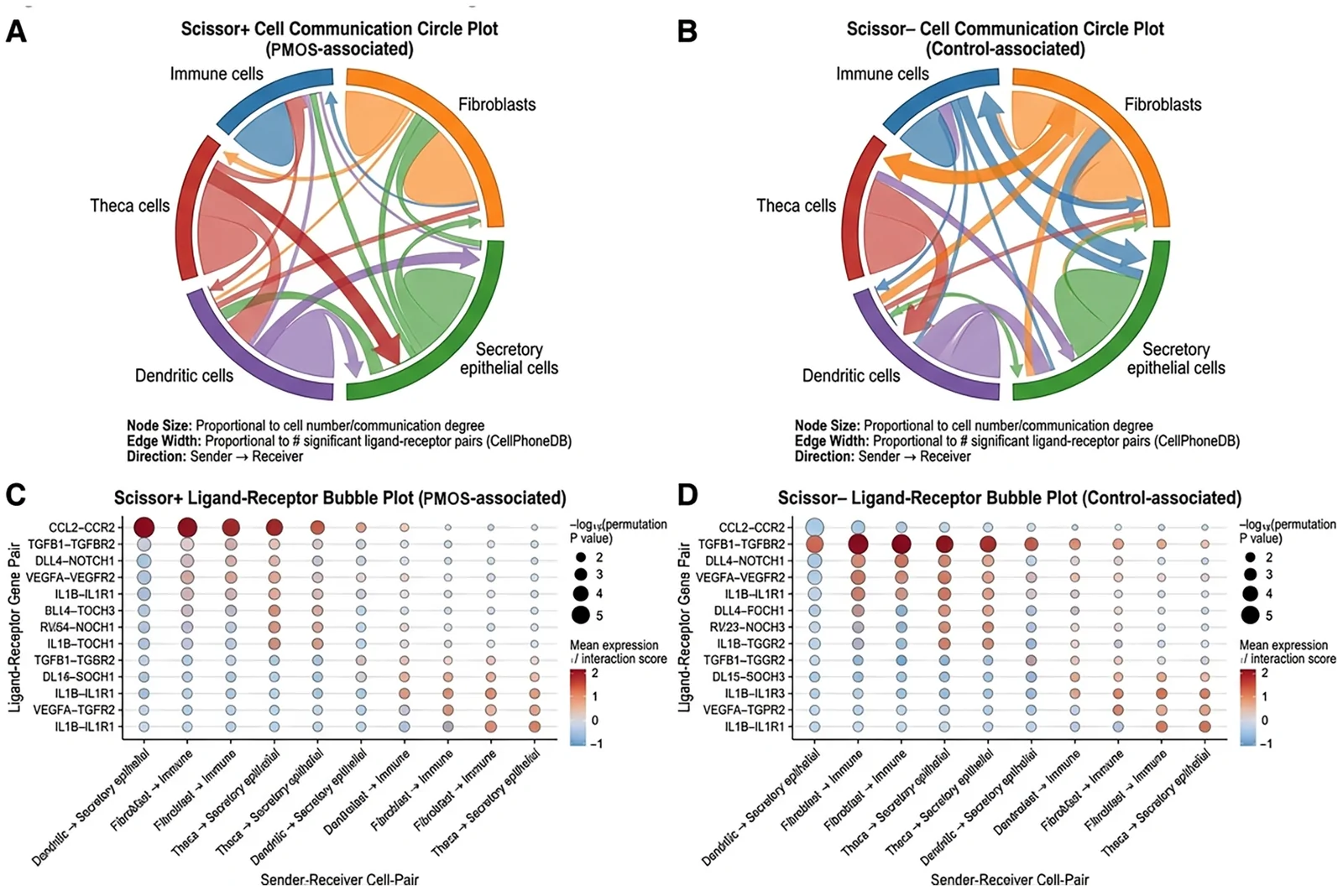
Predicted cell–cell communication analysis via CellPhoneDB comparing Scissor+ (polyendocrine metabolic ovarian syndrome (PMOS)-associated) and Scissor- (control-associated) cell states from PMOS ovarian single-cell RNA-sequencing (scRNA-seq) data. (**A**) Scissor+ interaction circle plot; (**B**) Scissor- interaction circle plot; (**C**) Scissor+ ligand-receptor bubble plot; (**D**) Scissor- ligand-receptor bubble plot. Communication outputs are computational predictions that require further experimental verification.

**Figure 9:**
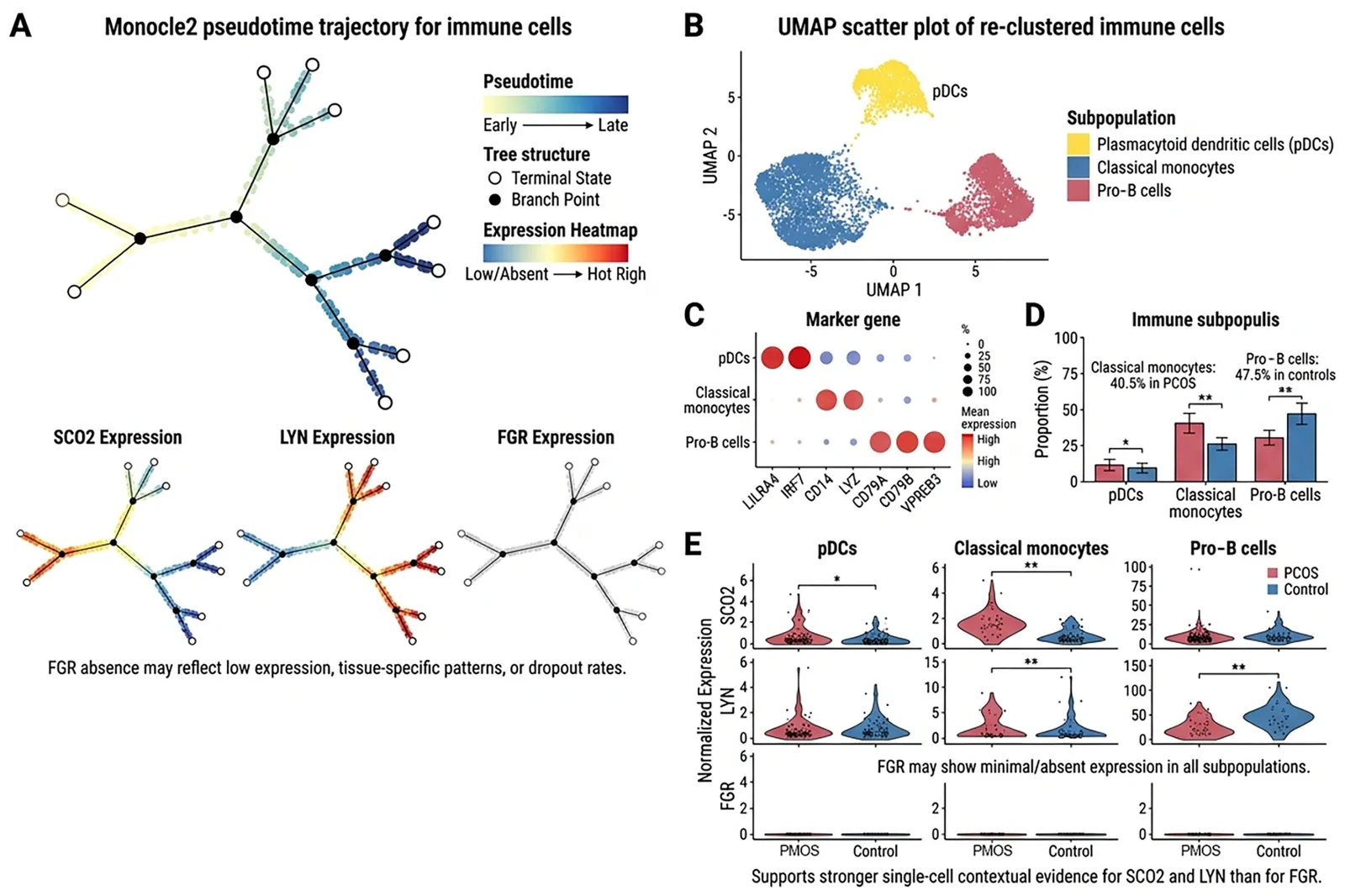
Pseudotime trajectory and immune-subpopulation re-clustering based on polyendocrine metabolic ovarian syndrome (PMOS) ovarian single-cell RNA-sequencing (scRNA-seq) data, exploring dynamic expression of SCO2, LYN, and FGR within immune-cell compartments. (**A**) Immune-cell pseudotime trajectory and SCO2/LYN/FGR expression; note that FGR was undetectable in this ovarian immune-cell dataset. (**B**) Uniform Manifold Approximation and Projection (UMAP) of re-clustered immune subpopulations. (**C**) Immune-subpopulation marker dot plot. (**D**) Immune-subpopulation proportions by group. (**E**) SCO2, LYN, and FGR expression across immune subpopulations. * *p* < 0.05, ** *p* < 0.01.

**Figure 10:**
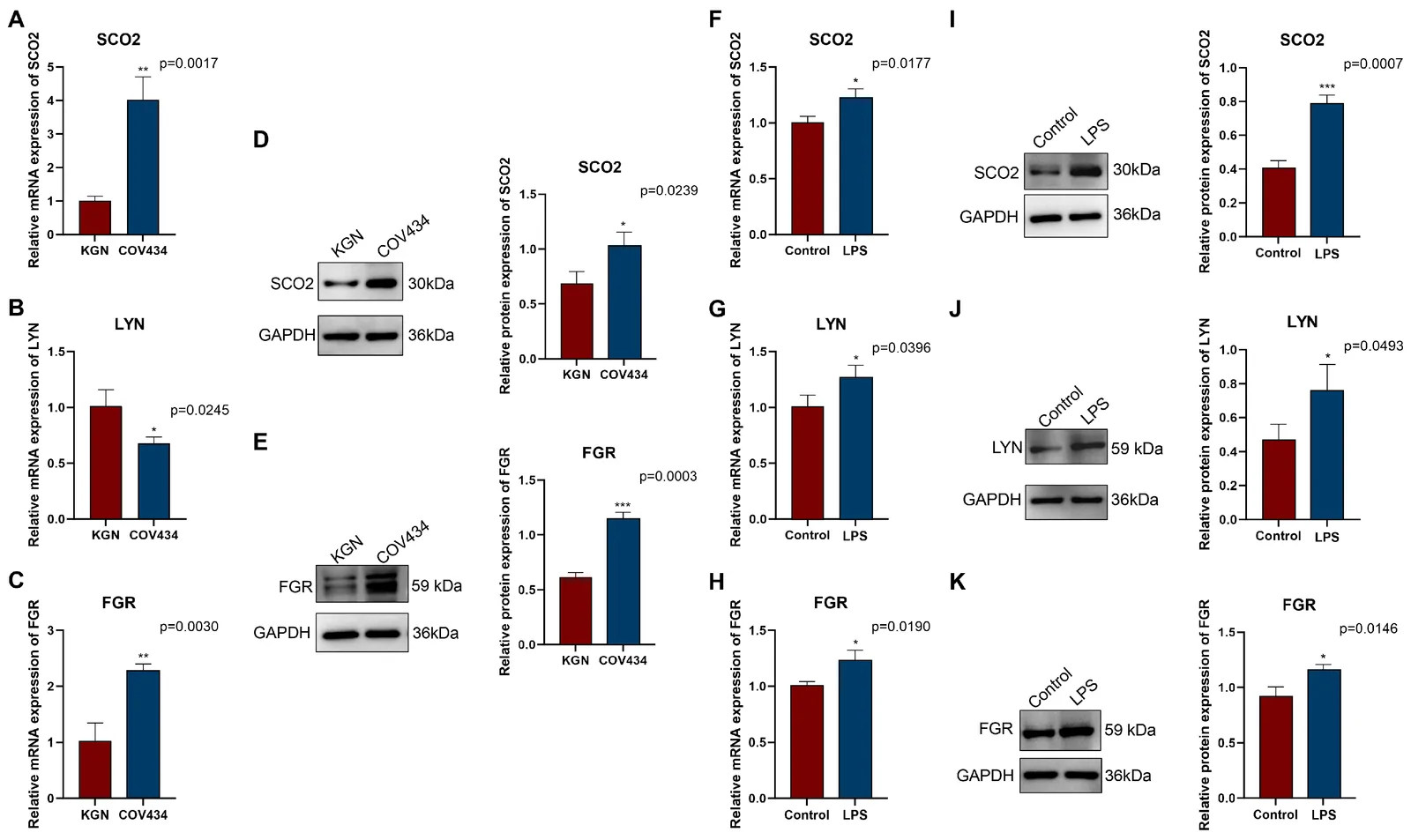
In vitro cell-model expression assessment of SCO2, LYN, and FGR. Two cell-model systems were used: COV434/KGN granulosa-cell lines for ovarian-cell-related changes; lipopolysaccharide (LPS)-treated BV2 microglia for general neuroinflammatory stress. (**A**–**C**) SCO2, LYN, and FGR mRNA expression in COV434 and KGN cells; (**D**,**E**) SCO2 and FGR protein expression in COV434 and KGN cells; (**F**–**H**) SCO2, LYN, and FGR mRNA expression in LPS-treated BV2 cells; (**I**–**K**) SCO2, LYN, and FGR protein expression in BV2 cells.* *p* < 0.05, ** *p* < 0.01, *** *p* < 0.001. Exact *p*-values are shown within each panel.

## Data Availability

PMOS and MCI data were retrieved from the Gene Expression Omnibus (GEO) database (http://www.ncbi.nlm.nih.gov/geo/, accessed on 5 August 2025). The single-cell RNA sequencing dataset for PMOS was downloaded from https://zenodo.org/records/7942968 (accessed on 5 August 2025). The MRGs were obtained from the MitoCarta 3.0 database (https://www.broadinstitute.org/mitocarta, accessed on 5 August 2025)). Detailed reporting frameworks, supplementary tables (S1–S4), supplementary figures (S1–S8), and a supplementary data checklist are provided in the Supplementary Material.

## Supplementary Materials

The following supporting information can be downloaded at: https://www.mdpi.com/article/10.3390/biomedicines14102205/s1, Figure S1: Differentially expressed genes (DEGs) sensitivity analysis; Figure S2: Feature-ranking reproducibility; Figure S3: Nomogram calibration details; Figure S4: Immune-signature sensitivity analysis; Figure S5: Single-cell RNA-sequencing (scRNA-seq) Quality Control (QC) and annotation diagnostics; Figure S6: Scissor classification and cell-state summaries; Figure S7: Predicted regulatory and docking evidence; Figure S8: Cell-line QC and raw-data reporting; Table S1: Descriptive statistics of Gene Expression Omnibus (GEO) datasets; Table S2: Basic physicochemical characterization of proteins; Table S3: Primer sequences; Table S4: Molecular docking SCO2-ampyrone binding energy (−4.8 kcal/mol) fell below the −5.0 kcal/mol threshold defined a priori.
