# Supplementary material for: SCO2, LYN, and FGR as Mitochondria-Associated Immune-Inflammatory Candidate Signatures Across PMOS and MCI Datasets
Source: Biomedicines. 2026 Sep 29;14(10):2205. doi: 10.3390/biomedicines14102205 (PMC13701576; doi:10.3390/biomedicines14102205)
Supplement: Supplementary file 1 [file biomedicines-14-02205-s001.zip › Figure legends Biomedicine.pdf]

Figure Legends

Supplementary Figure Legends

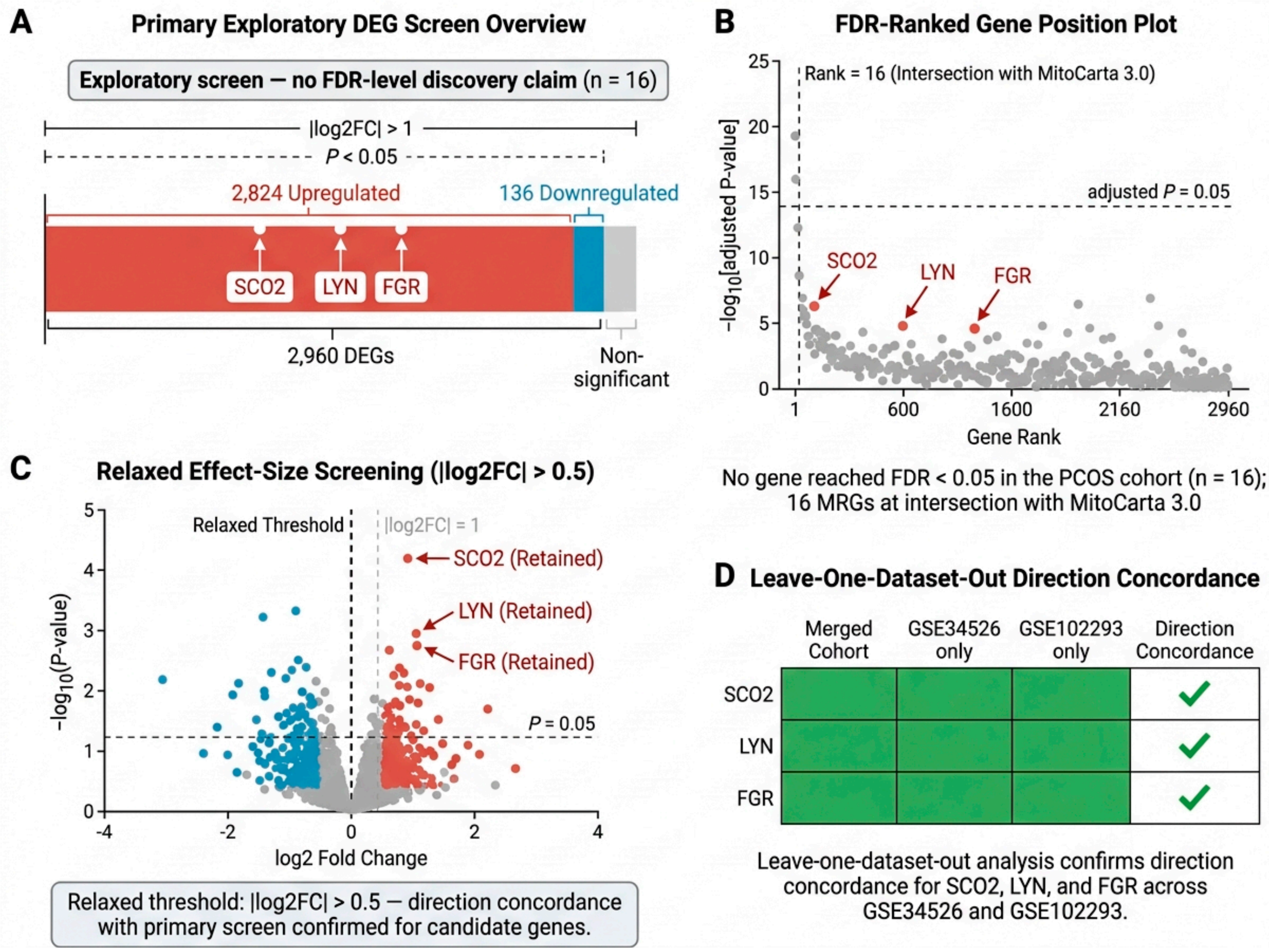

Supplementary Figure S1. DEG sensitivity analysis: (A) Primary PCOS DEG screen; (B) FDR-ranked exploratory DEG list; (C) Relaxed effect-size sensitivity screen; (D) Leave-one-dataset-out direction concordance.

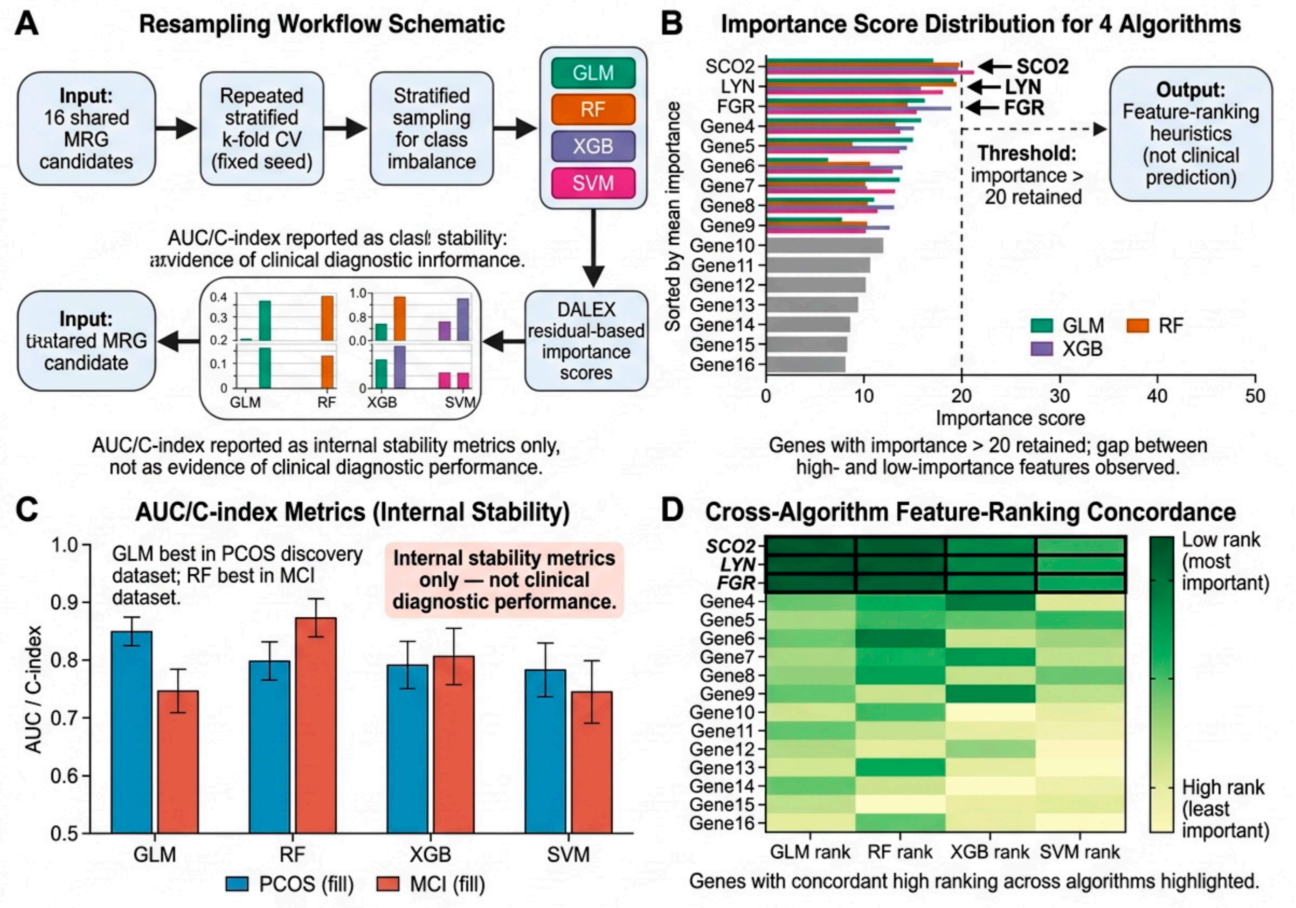

Supplementary Figure S2. Feature-ranking reproducibility: (A) Candidate-gene input set; (B) Resampling workflow; (C) Model-specific importance distributions; (D) Retained shared features.

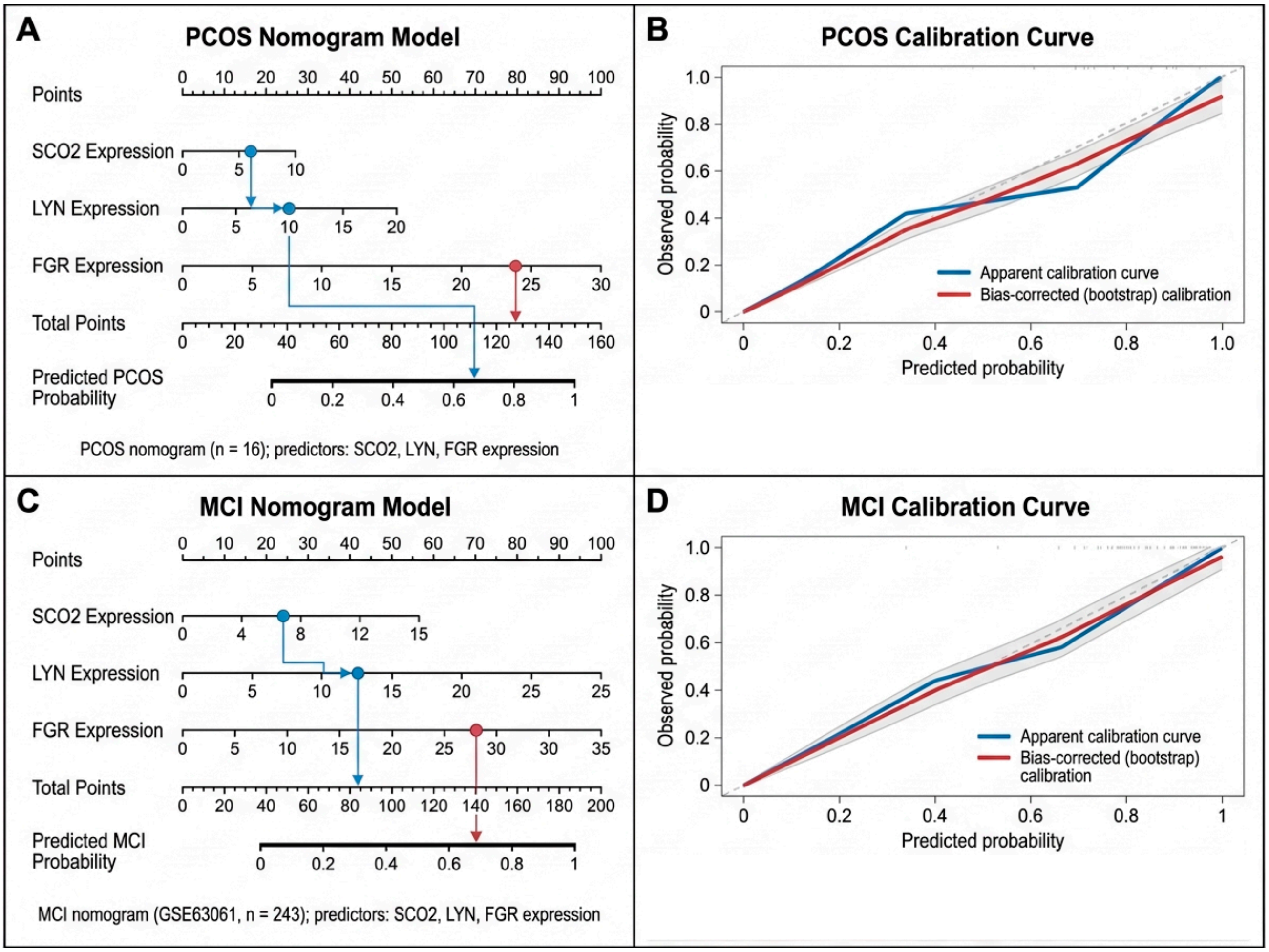

Supplementary Figure S3. Nomogram calibration details: (A) PCOS nomogram; (B) PCOS calibration curve; (C) MCI nomogram; (D) MCI calibration curve.

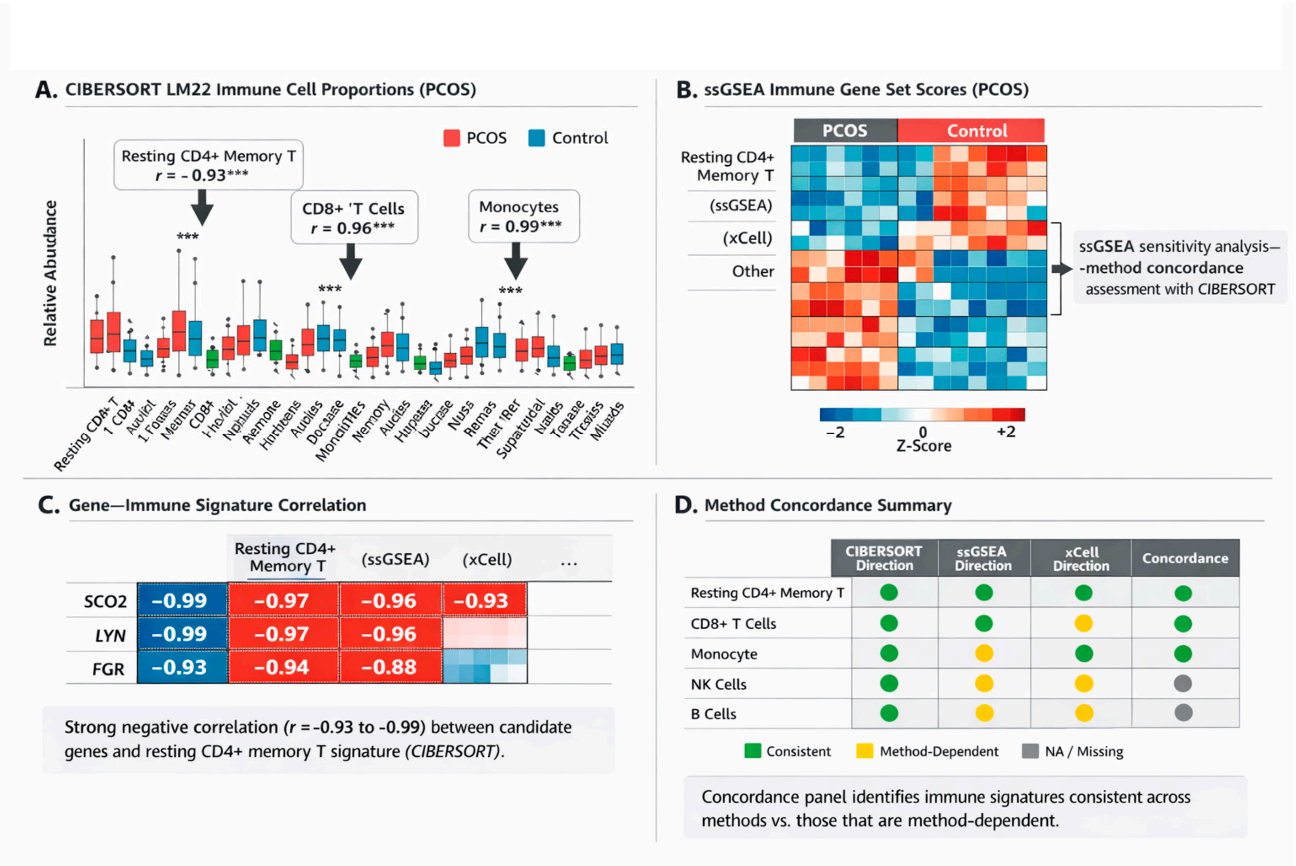

Supplementary Figure S4. Immune-signature sensitivity analysis: (A) CIBERSORT signatures; (B) ssGSEA/xCell immune scores; (C) Biomarker-immune correlations; (D) Cross-method concordance summary.

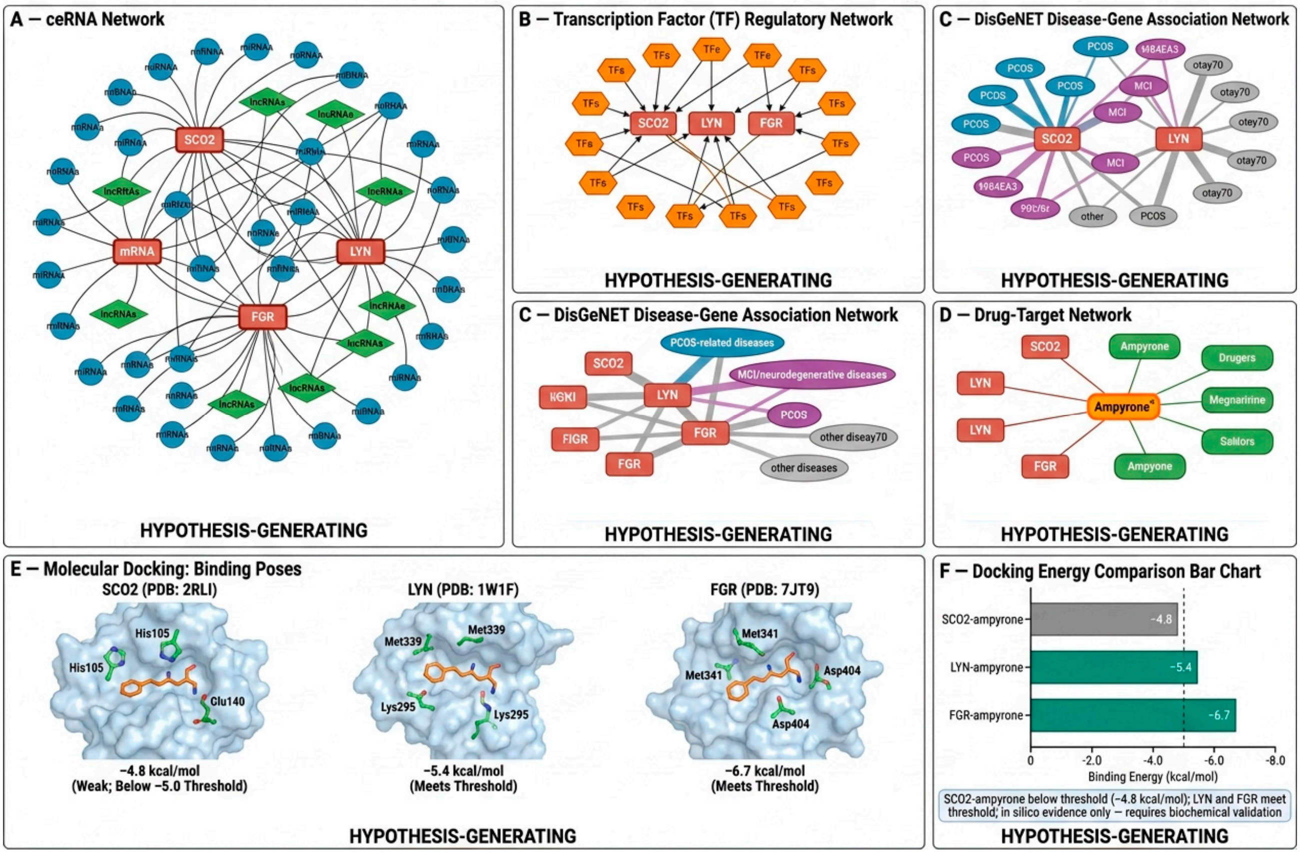

Supplementary Figure S5. scRNA-seq QC and annotation diagnostics: (A) Retained cells per donor; (B) Detected genes, UMI counts, and mitochondrial percentage; (C) Doublet-removal diagnostics; (D) Clustering and annotation markers.

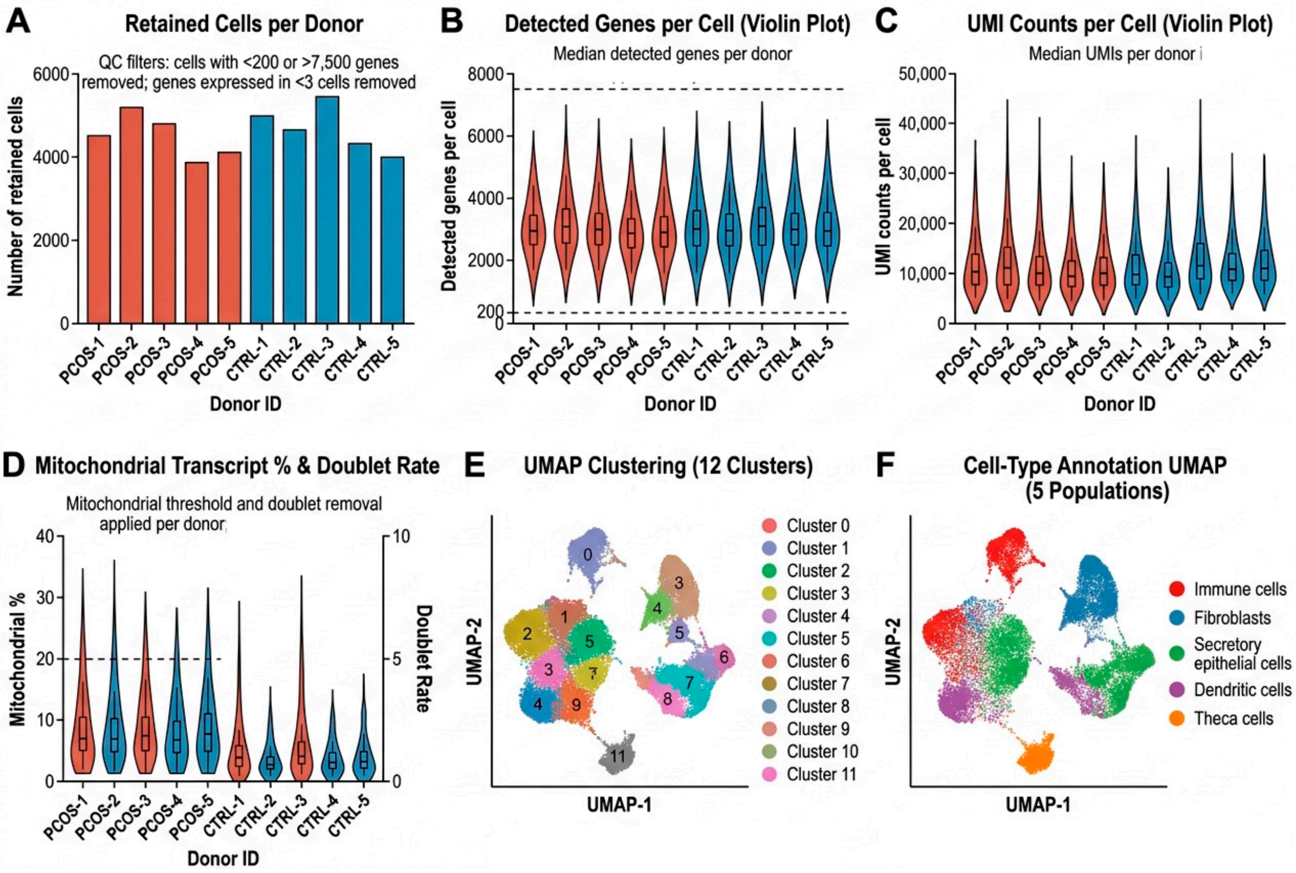

Supplementary Figure S6. Scissor classification and cell-state summaries: (A) Scissor classification on UMAP; (B) Scissor-defined cell counts by donor; (C) Donor-level cell-type proportions; (D) Candidate-gene expression along pseudotime.

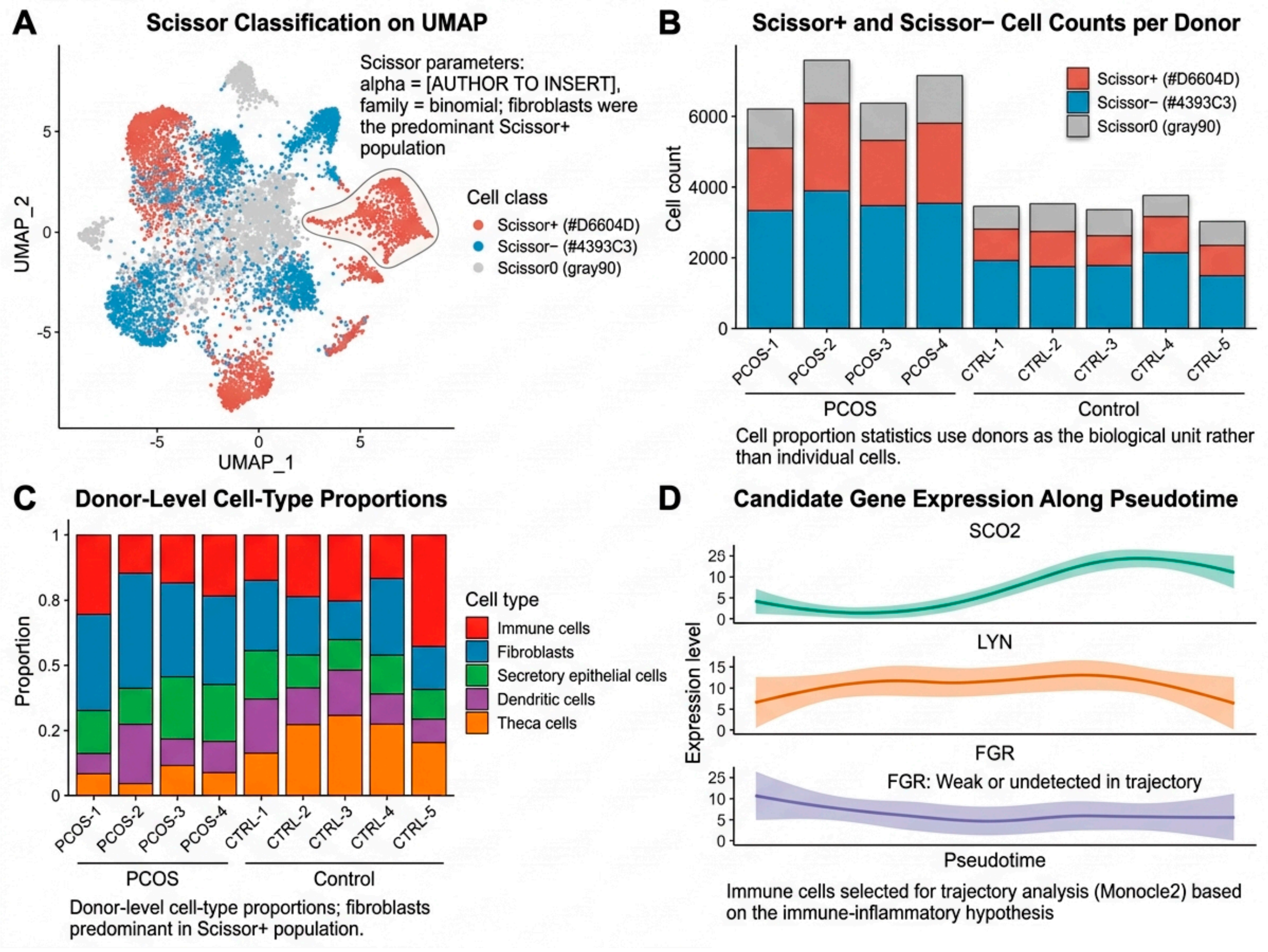

Supplementary Figure S7. Predicted regulatory and docking evidence: (A) ceRNA network; (B) TF-mRNA network; (C) Disease-biomarker network; (D) Drug-target network; (E-G) SCO2-, LYN-, and FGR-ampyrone docking poses.

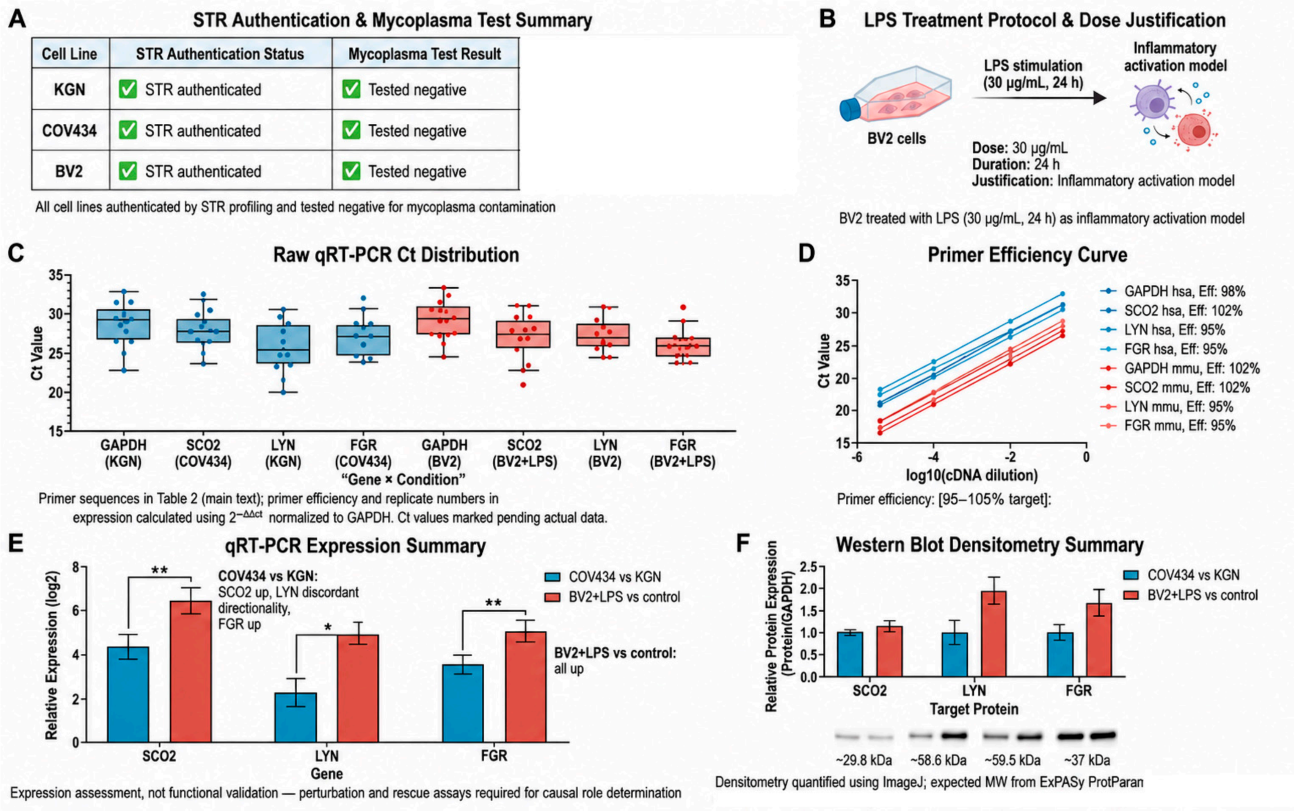

Supplementary Figure S8. Cell-line QC and raw-data reporting: (A) STR authentication and mycoplasma testing; (B) Passage range and LPS treatment scheme; (C) qRT-PCR Ct distribution and primer efficiency; (D) Uncropped western blots and densitometry workflow.

Abbreviations: ceRNA, competing endogenous RNA; DEG, differentially expressed gene; FDR, false discovery rate; GO, Gene Ontology; GSEA, Gene Set Enrichment Analysis; GSVA, Gene Set Variation Analysis; KEGG, Kyoto Encyclopedia of Genes and Genomes; MCI, mild cognitive impairment; PCOS, polycystic ovary syndrome; scRNA-seq, single-cell RNA sequencing; TF, transcription factor; UMAP, Uniform Manifold Approximation and Projection.
