# Supplementary material for: SCO2, LYN, and FGR as Mitochondria-Associated Immune-Inflammatory Candidate Signatures Across PMOS and MCI Datasets
Source: Biomedicines. 2026 Sep 29;14(10):2205. doi: 10.3390/biomedicines14102205 (PMC13701576; doi:10.3390/biomedicines14102205)
Supplement: Supplementary file 1 [file biomedicines-14-02205-s001.zip › Supplementary Materials-final.pdf]

Supplementary Methods

Sensitivity analysis of PCOS DEG selection. The primary PCOS DEG screen is retained as exploratory because of the small sample size (n = 16). The supplementary analysis reports FDR-ranked candidate positions, relaxed effect-size screening (|log2FC| > 0.5), and leave-one-dataset-out direction concordance for GSE34526 and GSE102293. The PCOS discovery cohort contained 2,960 DEGs (2,824 upregulated, 136 downregulated) at |log2FC| > 1 and nominal P < 0.05.

Machine-learning reproducibility. The candidate-gene models are documented as feature-ranking procedures. A fixed random seed was set for all four algorithms (GLM, RF, XGB, SVM). Repeated stratified k-fold cross-validation was used, with class imbalance addressed through stratified sampling. Genes with importance scores > 20 were retained. The feature-ranking procedure, including fold number, repeat number, class-balance handling, and tuning configuration. Model outputs are presented as feature-ranking heuristics rather than validated predictive models.

Nomogram calibration. Nomograms are presented as exploratory disease classifiers. Calibration curves were assessed using bootstrap resampling. The calibration slope, intercept, C-index/AUC, and 95% confidence intervals for both nomogram models are reported in Supplementary Table S5. Given the limited PCOS sample size (n = 16) and the absence of a clinically confirmed PCOS-MCI comorbidity cohort, these models were exploratory disease-classification tools. No external PCOS-MCI validation cohort was available.

Immune-signature sensitivity. CIBERSORT outputs for PCOS granulosa-cell datasets are described as immune-related transcriptional signatures rather than immune-cell infiltration, because LM22 was developed for blood immune cell mixtures. ssGSEA and/or xCell sensitivity analyses are summarized to evaluate method concordance. Because PCOS datasets are derived from granulosa cells rather than immune-rich tissues, results reflect immune-related transcriptional signatures.

Single-cell reporting. Donor-level QC, doublet rate (DoubletFinder), mitochondrial

transcript threshold (<200 or >7,500 detected genes excluded; genes in <3 cells removed), integration parameters, clustering resolution, marker genes, Scissor settings, and donor-level cell proportion statistics are reported. The PCOS scRNA-seq dataset identified 12 clusters annotated into five populations: immune cells, fibroblasts, secretory epithelial cells, dendritic cells, and theca cells (Supplementary Tables S7-S9).

Prediction-level networks and docking. ceRNA, transcription factor (TF), DisGeNET, drug-target, and docking analyses are condensed into supplementary evidence and described as hypothesis-generating. Molecular docking was performed using AutoDock Vina with PDB structures 2RLI (SCO2), 1W1F (LYN), and 7JT9 (FGR) against ampyrone as ligand. Binding energies were -4.8, -5.4, and -6.7 kcal/mol respectively (Supplementary Table S13). The -5.0 kcal/mol threshold was defined a priori; SCO2-ampyrone fell below this threshold.

Cell-line expression-assessment reporting. STR authentication, mycoplasma testing, passage range, reagent details, raw Ct values, uncropped western blot membranes, antibody records, and biological/technical replicate definitions are provided. KGN, COV434, and BV2 cell lines were authenticated by STR profiling and tested negative for mycoplasma (Supplementary Tables S10-S12). BV2 cells were treated with LPS (30 ug/mL, 24 h) as an inflammatory activation model. Expression was calculated using the 2^-ddCt method normalized to GAPDH.

Table S1. Descriptive statistics of Gene Expression Omnibus (GEO) datasets

Disease	Types of datasets	GEO accession	Platform	Origin	Sample (total)	Disease	Control	Species
PCOS	Training dataset	GSE34526	GPL570	granulosa cells	10	7	3	Homo sapiens
		GSE102293	GPL570	granulosa cells	6	2	4	Homo sapiens

	Validation dataset	GSE137684	GPL17077	granulosa cells	12	8	4	Homo sapiens
MCI	Training dataset	GSE63061	GPL10558	Blood	243	109	134	Homo sapiens
	Validation dataset	GSE63060	GPL6947	Blood	184	80	104	Homo sapiens

Abbreviation: PCOS, Polycystic Ovary Syndrome; MCI, mild cognitive impairment

Table S2. Basic physicochemical characterization of proteins

Physicochemical characterization	SCO2	LYN	FGR
Number of amino acids	266	512	529
Theoretical pI	9.01	6.70	5.41
Molecular weight	29809.94	58574.09	59478.62
Formula	$C_{1325}H_{2084}N_{392}O_{380}S_7$	$C_{2634}H_{4113}N_{695}O_{774}S_{21}$	$C_{2663}H_{4101}N_{707}O_{791}S_{25}$
Estimated half-life	30 hours	30 hours	30 hours
Instability index	43.97	37.21	42.00
Aliphatic index	85.83	79.04	77.11
Grand average of hydropathicity	-0.400	-0.503	-0.358

Table S3 Primer sequences (F = forward, R = reverse)

Primer	Sequence 5'-3'
hsa-GAPDH	F:5‘-CTGGGCTACACTGAGCACC-3’
	R:5‘-AAGTGGTCGTTGAGGGCAATG-3’
hsa-SCO2	F:5‘-CTTCCTCTCGTGCTTGGTCC-3’
	R:5‘-AAAGCCAGGACCTCAGATGC-3’

hsa-LYN	F:5‘-TAAACAGCAAAGGCCAGTTCC-3’
	R:5‘-AAAGACAAGTCGTCCGGGTG-3’
hsa-FGR	F:5‘-AATTGGGCTCCAGGTCTCTG-3’
	R:5‘-CACCGGCTCCAATTTCTTGC-3’
mmu-GAPDH	F:5‘-AGGTCGGTGTGAACGGATTTG-3’
	R:5‘-TGTAGACCATGTAGTTGAGGTCA-3’
mmu-SCO2	F:5‘-TCCCGATCTTTCCTGGTCCT-3’
	R:5‘-CTCGGACGCACTCAACAATG-3’
mmu-LYN	F:5‘-TTCCACCACGAGCGAGAAAT-3’
	R:5‘-GGAACTGGCCTTTGCTGTTT-3’
mmu-FGR	F:5‘-CTGTTGAATGCTCACCACCC-3’
	R:5‘-GTTCGCCTCCTGCAGACTAC-3’

Table S4 Molecular docking †SCO2-ampyrone binding energy (-4.8 kcal/mol) fell below the -5.0 kcal/mol threshold defined a priori. This interaction is presented for completeness and should be interpreted as a hypothesis-generating experiment only.†

Gene Symbol	PDB	Chemical Name	kcal/mol
SCO2	2RLI	ampyrone	-4.8
LYN	1W1F	ampyrone	-5.4
FGR	7JT9	ampyrone	-6.7
